# Guanidines: Privileged Scaffolds Against Neglected Tropical Diseases: A Review

**DOI:** 10.3390/ph19050784

**Published:** 2026-05-17

**Authors:** Luana Ribeiro dos Anjos, Rodrigo Santos Aquino de Araújo, Malu Maria Lucas dos Reis, Natalia C. S. Costa, Vitória Gaspar Bernardo, Eduardo Henrique Zampieri, Klinger Antonio da Franca Rodrigues, Eduardo Maffud Cilli, Eduardo René Pérez González, Francisco Jaime Bezerra Mendonça-Junior

**Affiliations:** 1 Fine Organic Chemistry Laboratory School of Sciences and Technology, São Paulo State University (UNESP), Presidente Prudente 19060-080, São Paulo, Brazil; luana.anjos@unesp.br (L.R.d.A.); eduardo.h.zampieri@unesp.br (E.H.Z.); 2 Federal Institute of Education, Science, and Technology os Sertão Pernambucano (IFSertãoPE), Campus Santa Maria da Boa Vista, Santa Maria da Boa Vista 56380-000, Pernambuco, Brazil; 3 Laboratory of Synthesis and Drug Delivery, Department of Biological Sciences, State University of Paraíba, João Pessoa 58071-160, Paraíba, Brazil; malureisduarte@gmail.com (M.M.L.d.R.); vit.gaspar.bernardo@gmail.com (V.G.B.); 4 Department of Biochemistry and Organic Chemistry, Institute of Chemistry, São Paulo State University (UNESP), Araraquara 14800-060, São Paulo, Brazil; natalia.costa@unesp.br (N.C.S.C.); eduardo.cilli@unesp.br (E.M.C.); 5 Post-Graduate Program in Therapeutic Innovation, Biological Sciences Center, Federal University of Pernambuco, Recife 50670-420, Pernambuco, Brazil; 6 Infectious Disease Laboratory—LADIC, Federal University of Parnaíba Delta—UFDPar, Campus Ministro Reis Velloso, São Benedito, Parnaíba 64202-020, Piauí, Brazil; klinger.antonio@gmail.com

**Keywords:** guanidine derivatives, neglected diseases, medicinal chemistry, structure–activity relationship, drug discovery, antiparasitic compounds

## Abstract

**Background:** Neglected diseases caused by protozoan parasites remain a major public health burden, particularly in low- and middle-income countries. Among the chemical motifs explored in antiparasitic drug discovery, guanidine-containing compounds have attracted considerable attention due to their strong cationic character, high capacity for hydrogen bonding, and versatility in interacting with biological targets. **Methodology:** This review summarizes advances reported in the last decade regarding guanidine derivatives with activity against pathogens associated with Chagas disease, human African trypanosomiasis, Leishmaniasis, tuberculosis, toxoplasmosis, dengue and schistosomiasis. **Results:** Evidence gathered from synthetic, natural, and drug-repurposing studies indicates that the guanidine, guanidine-containing and guanidine-related compounds contribute to modulating biological activity by changing electrostatic interactions, hydrogen-bonding networks, and physicochemical properties, with enzymes, nucleic acids, and membrane-associated targets essential for parasite survival. Across the analyzed studies, several emerging structure–activity relationship trends were identified, including the contribution of polycationic or dicationic architectures, the influence of halogenated or lipophilic substituents, and the dependence of biological activity on the complete molecular framework, including heterocyclic systems, macrocycles, peptide conjugates, hybrid scaffolds, and repurposed drugs. In addition to direct antiparasitic effects, certain guanidine-containing and guanidine-related compounds demonstrate immunomodulatory or host-protective properties, expanding the therapeutic relevance of this class. Despite promising in vitro results, protonation trapping, efflux pump susceptibility, and pharmacokinetic limitations such as poor oral absorption, high polarity, plasma protein binding and limited membrane permeability remain significant challenges for clinical translation. Nonetheless, the integration of medicinal chemistry, computational modeling, and biological screening continues to accelerate the identification of optimized scaffolds. **Conclusions:** Overall, guanidine-based compounds constitute a promising scaffold for the development of new therapeutic strategies targeting neglected parasitic diseases, and further structural optimization may enable the emergence of candidates with improved efficacy, selectivity, and drug-like properties.

## 1. Introduction

Neglected tropical diseases (NTDs) are a heterogeneous group of infectious conditions that disproportionately affect populations in low- and middle-income countries where access to healthcare, sanitation, and effective pharmacological treatments is limited. These diseases are caused by a broad range of pathogens, including parasites, bacteria, viruses and fungi, and frequently involve complex transmission cycles mediated by vectors or animal reservoirs. Despite their significant health and socioeconomic impact, NTDs have historically received limited investment in research and development (R&D), largely due to the low commercial incentives for the pharmaceutical industry. As a result, the therapeutic arsenal available for many of these infections remains scarce and inadequate, often relying on old drugs characterized by limited efficacy, toxicity, restricted efficacy in chronic stages of infection, or emerging resistance [1,2,3,4,5,6].

**Figure 1:**
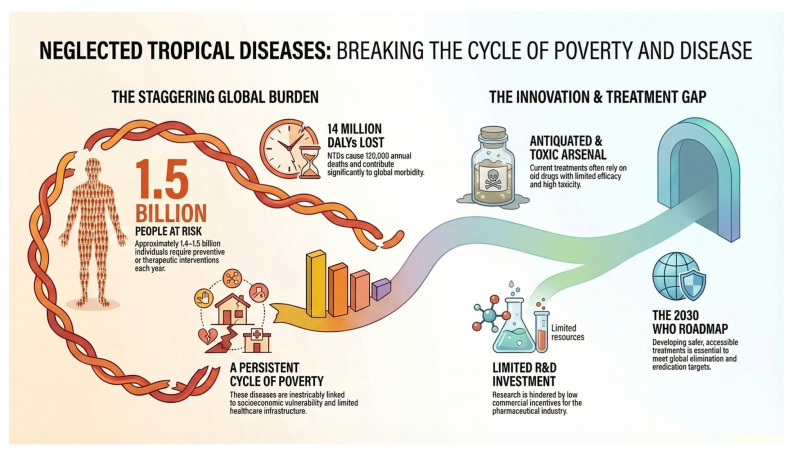
Schematic overview of neglected tropical diseases and their global relevance. The information summarized in the figure is based on the epidemiological sources cited in the Introduction and is intended as a conceptual representation rather than a primary quantitative dataset.

From a global health perspective, NTDs constitute a major burden, affecting more than one billion people worldwide, with approximately 1.4–1.5 billion individuals requiring preventive or therapeutic interventions each year. The number of annual deaths and of disability-adjusted life years (DALYs) varies depending on the source of information and on the number of diseases considered in the study, but there is a consensus that collectively, these diseases are responsible for more than 100,000 deaths annually and between 12 and 20 million DALYs, underscoring their substantial contribution to global morbidity. Their epidemiology is strongly associated with socioeconomic vulnerability, environmental factors, and limited healthcare infrastructure, conditions that perpetuate a persistent cycle of poverty and disease in endemic regions (Figure 1) [2,3,4,5,6].

The World Health Organization (WHO) recognizes a group of NTDs, which include Chagas disease, human African trypanosomiasis, leishmaniasis, lymphatic filariasis, schistosomiasis, onchocerciasis, leprosy, rabies, and soil-transmitted helminthiases, among others. Arboviral infections such as dengue and, in some contexts, chikungunya, are also frequently discussed within the broader framework of neglected or emerging diseases affecting vulnerable tropical and subtropical populations. These diseases represent critical challenges for medicinal chemistry and drug discovery, since the development of safer, more effective, and accessible treatments remains essential to achieving the WHO roadmap goals for control, elimination, or eradication of NTDs by 2030 (Figure 1) [1,2].

Within this context, medicinal chemistry plays a central role in expanding the pipeline of candidate compounds for NTDs through strategies such as ligand-based and structure-based drug design, high-throughput screening, and rational scaffold optimization [7,8,9,10,11]. In addition to *de novo* drug discovery, drug repurposing has emerged as an attractive complementary approach, as it allows the identification of new therapeutic applications for existing compounds with previously characterized pharmacokinetic and safety profiles, thereby reducing development time, cost, and attrition rates [12,13,14,15,16]. Such strategies have proven particularly valuable for neglected diseases, where limited funding and market incentives restrict traditional drug discovery efforts.

Among the various chemical scaffolds investigated for antiparasitic drug discovery, guanidine-containing compounds have attracted increasing attention due to their versatile physicochemical and biological properties [17,18,19,20]. The guanidine moiety is characterized by strong basicity, high nucleophilicity, and the capacity to establish multiple hydrogen-bonding and electrostatic interactions with biological targets, which can contribute to binding interactions toward enzymes, proteins, and nucleic acids [21,22]. In addition to their biological relevance, guanidines are also valuable synthetic building blocks and can be incorporated into diverse structural frameworks, including heterocycles, acyclic derivatives, polymeric systems, and natural products, allowing extensive chemical diversification and fine modulation of physicochemical and pharmacological properties. This synthetic versatility supports their use in medicinal chemistry programs aimed at generating structurally diverse heterocycles and hybrid scaffolds for antiparasitic drug discovery [23,24,25,26].

**Figure 2:**
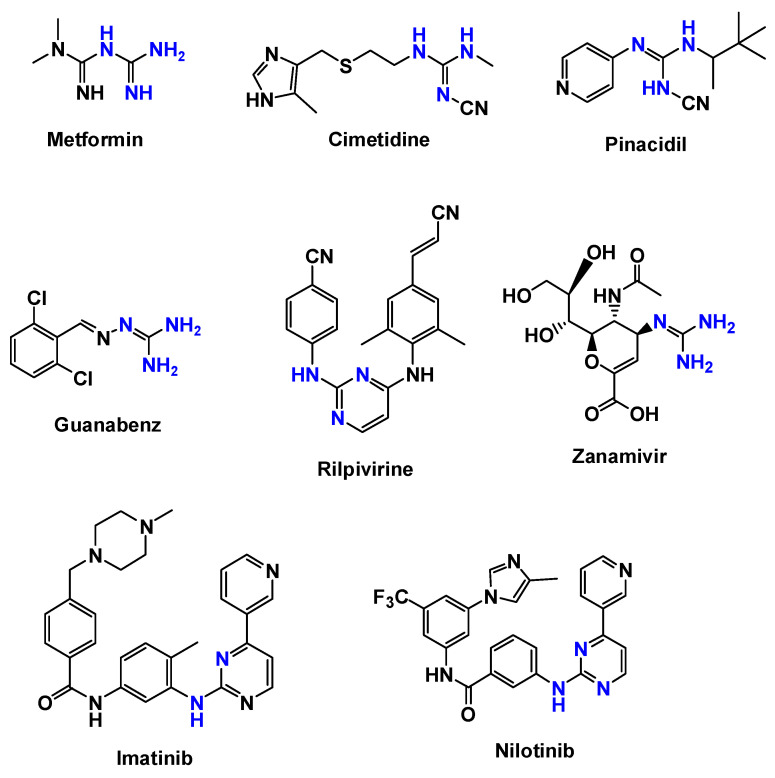
Example of commercial drugs containing guanidine, guanidine-containing and guanidine-related compounds, including amidines, and biguanides.

Historically, guanidine, guanidine-containing and guanidine-related compounds, including amidine and biguanide motifs, have been associated with numerous biologically active molecules, including antimicrobial agents, antiparasitic drugs, and enzyme inhibitors [27,28,29,30,31,32,33,34,35,36]. Notably, the guanidine motif (R_2_N-(C=NR)-NR_2_) is also present in the chemical structure of several clinically commercial drugs across different pharmacological classes. Representative examples include the antidiabetic metformin (a biguanide), the histamine H_2_ receptor antagonist cimetidine, the antihypertensives pinacidil and guanabenz, the antivirals rilpivirine and zanamivir, and the kinase inhibitors used to treat cancer, imatinib and nilotinib (Figure 2). The presence of guanidinium functionality in these therapeutics highlights its pharmacological versatility and its ability to engage in strong electrostatic and hydrogen-bonding interactions with biological targets.

In the field of trypanosomatid research, for example, some cationic compounds containing guanidinium or related functionalities have been investigated for their ability to interact with parasite DNA, interfere with polyamine-associated metabolism, and inhibit key enzymatic pathways [7,37,38]. Similarly, selected guanidine-containing or guanidine-related compounds have been explored either as direct antiparasitic agents or as components of control strategies targeting vectors or intermediate hosts (vector/ecological control approaches) [39,40]. These observations have stimulated growing interest in the rational design and evaluation of guanidinic molecules as potential therapeutic agents against neglected parasitic diseases, particularly within medicinal chemistry programs focused on the discovery and optimization of small-molecule antiparasitic agents [8,17,18,39,41,42].

Over the past decade, advances in medicinal chemistry, computational modeling, high-throughput screening, and drug repurposing strategies have accelerated the identification of guanidine-based compounds with promising antiparasitic profiles. Importantly, the accumulation of biological data has enabled the identification of emerging structure–activity relationship (SAR) trends, highlighting how electronic and steric effects, substitution patterns, cationic character, and molecular flexibility influence antiparasitic potency and selectivity. At the same time, these studies have revealed recurring pharmacokinetic challenges associated with highly basic molecules, including protonation trapping, efflux pump susceptibility, limited membrane permeability, limited plasma protein binding, and suboptimal bioavailability, which remain important considerations for the development of clinically viable candidates [43,44,45,46].

Given the expanding body of literature in this field, a comprehensive synthesis of recent findings is both timely and necessary. In this context, the present review summarizes and critically analyzes advances reported between 2015 and 2025 concerning guanidine-containing and guanidine-related compounds with activity against pathogens associated with major neglected tropical diseases (NTDs). Throughout this review, the terms guanidine, guanidinium, guanidino, biguanide, amidine, nitroguanidine, etc., are used according to their structural meaning. When compounds contain related but non-identical nitrogen-rich motifs, they are discussed as guanidine-related or amidine/biguanide-related compounds rather than as classical guanidine derivatives. Particular emphasis is placed on medicinal chemistry strategies employed in the design and synthesis of these molecules, their biological evaluation, proposed mechanisms of action, and emerging structure–activity relationship (SAR) trends that may inform future drug discovery efforts. By integrating evidence from synthetic derivatives, natural products, and repositioned drugs, this review provides a coherent overview of how the guanidine motif continues to contribute to the identification of new antiparasitic agents and highlights perspectives for future research in this area.

## 2. Results and Discussion

### 2.1. Guanidine Derivatives Targeting Trypanossoma cruzi

Chagas disease is caused by the protozoan *Trypanosoma cruzi* and remains one of the most relevant trypanosomatid infections worldwide. More than 25 million individuals live in areas at risk of infection, with approximately 7 million infected globally, and the disease is considered by the WHO to present a significant mortality risk [47]. Transmission occurs mainly through contact with the feces or urine of infected triatomine insects during blood feeding [48]. Although historically concentrated in Latin America, population mobility has resulted in cases being reported in regions such as Europe and Japan [49].

**Figure 3:**
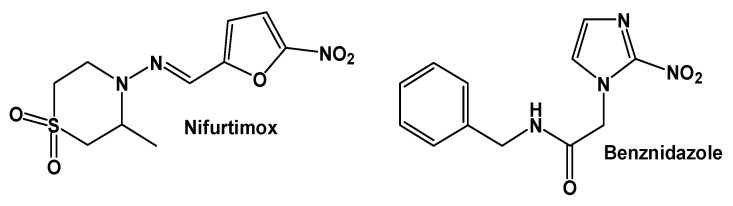
Chemical structure of the commercial drugs Nifurtimox and Benznidazole, used in the treatment of Chagas disease.

Clinically, the disease may progress to severe chronic manifestations, including cardiomegaly and megacolon in approximately 30% of patients [50]. Diagnosis often occurs during the chronic phase, further complicating treatment [51]. Current chemotherapy relies essentially on nifurtimox and benznidazole (Figure 3), both associated with adverse effects and limited efficacy in chronic infection, which underscores the urgent need for alternative therapeutic strategies [52,53]. In parallel, vector control programs have contributed to reducing transmission in some regions, although the emergence of insecticide resistance among triatomines has renewed interest in identifying new prophylactic approaches [54,55].

Drug repurposing has emerged as a valuable strategy to accelerate the identification of new antiparasitic agents [56,57]. In this context, Juarez-Saldivar et al. [58] performed a virtual screening of FDA-approved drugs targeting *T. cruzi* triosephosphate isomerase (*Tc*TIM), an essential enzyme in glucose metabolism whose parasite homolog differs significantly from the human enzyme [59].

**Figure 4:**
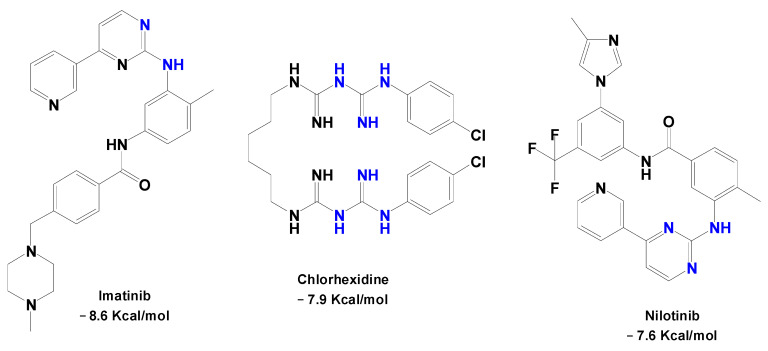
Selected commercial drugs containing guanidine moiety with their respective affinity values against the *T. cruzi* triosephosphate isomerase enzyme (*Tc*TIM) (PDB id 1SUX).

Using a structure-based docking approach with the crystal structure of *Tc*TIM (PDB id 1SUX), a library of 1467 approved drugs was screened, leading to the selection of eight candidates for in vitro evaluation. Among these, three compounds contained guanidine moieties in their structures: imatinib, chlorhexidine, and nilotinib. Docking studies suggested possible binding modes within the active site of *Tc*TIM, including hydrophobic contacts with Tyr102 (B), Ile109 (A), and Phe75 (A), π-stacking interactions involving Tyr103 (B) and Phe75 (A), and hydrogen bonds with Tyr102 (B). Although these residues are known to play a role in binding the ligand to TcTIM, these computational results should be interpreted as hypotheses for potential target engagement and not as direct evidence of binding or as quantitative predictors of the observed biological activity [60]. Among the guanidine-containing molecules, chlorhexidine displayed the most extensive interaction network, forming four hydrogen bonds with Tyr102 (B) and Thr70 (A). Figure 4 shows the chemical structures of these compounds along with their respective affinity values obtained in the protein-ligand interactions.

Subsequent in vitro assays evaluated trypomastigote lysis against INC-5 and NINOA strains of *T. cruzi*, as well as cytotoxicity in murine macrophages (J774.A1). Chlorhexidine exhibited the highest activity among the guanidine derivatives, with LC_50_ values of 23.10 and 9.69 µM for the INC-5 and NINOA strains, respectively, representing a potency approximately 11–22 times greater than benznidazole and nifurtimox. However, the compound showed moderate cytotoxicity, with a CC_50_ value of 39.85 µM. The activity profile was attributed not only to the multiple hydrogen-bond donor sites present in chlorhexidine, but also to its cationic bis-biguanide architecture, which may favor electrostatic interactions with negatively charged biological surfaces, membrane-associated disruption mechanisms, and/or stabilizing interactions with enzymatic targets [61].

Arginase has also been investigated as a potential molecular target in trypanosomatid parasites [62]. This enzyme catalyzes the conversion of arginine to ornithine in the final step of the urea cycle [63]. Ornithine subsequently serves as a precursor for polyamine biosynthesis in non-hepatic tissues [64]. Arginine is also a substrate for nitric oxide synthase (NOS), which plays an important role in immune response modulation [65]. Trypanosomatid parasites such as *Trypanosoma cruzi* and *Trypanosoma brucei* appear to upregulate arginase expression and activity, leading to reduced arginine availability and consequently decreased nitric oxide production. The regulation of this process involves multiple and complex pathways, which have the potential to weaken host immune responses while simultaneously promoting polyamine synthesis, which is essential for parasite growth and differentiation [66]. Therefore, the development of arginase inhibitors represents a promising strategy in the search for novel antitrypanosomal agents.

**Figure 5:**
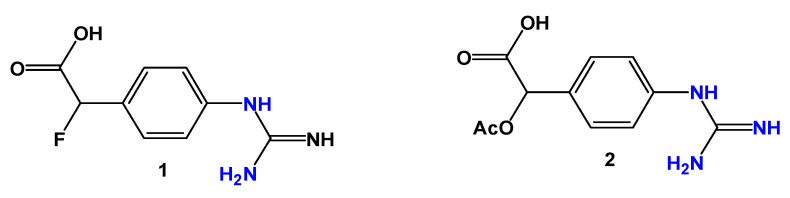
Chemical structures and synthetic approach of the proposed arginase inhibitors.

Based on known arginase inhibitors, Guo, Chen, and Seto [66] identified three key structural regions: a 4-guanidinobenzyl fragment that mimics the side chain of *L*-arginine; a leaving group capable of interacting with nucleophilic residues in the arginase active site; and a carboxylate group responsible for hydrogen bonding within the catalytic pocket. Guided by these features, two inhibitors (**1** and **2**, Figure 5) were designed and initially evaluated through molecular docking studies using the crystal structure of the arginase–ABH complex (ABH: an arginine boronic acid analog; PDB ID: 2AEB) and the AutoDock Vina software version 1.2.0.

Docking studies indicated that compound **1** exhibited a binding mode similar to that of the reference inhibitor ABH, forming hydrogen bonds between its guanidinium group and residues His126, Asp128, Asp232, and Asp234, as well as interactions between the carboxylate group and residues Asn130, Ser137, and Asn139. Compound **2** displayed a comparable binding orientation but with fewer favorable interactions.

The inhibitory activity of compounds **1** and **2** was evaluated through arginase catalytic assays using a DTNB (5,5-dithio-*bis*-(2-nitrobenzoic acid)) colorimetric method with thioarginine as substrate. The inhibition constants obtained for compound **1** (Ki = 275 and 322 µM for arginase I and II, respectively) and compound 2 (Ki = 382 and 437 µM) were substantially lower than those observed for the substrate thioarginine (1.4 and 1.5 mM), likely due to the increased hydrophobicity of the inhibitors. Compound **1** exhibited slightly improved inhibitory potency compared with compound **2**, in agreement with the docking results. Further mechanistic studies demonstrated that both inhibitors act as irreversible arginase inactivators through covalent modification of residue Thr135. These findings provide valuable insights for the design of improved arginase inhibitors with potential antitrypanosomal activity [66].

Some guanidine-containing derivatives, particularly aromatic or polycationic molecules with suitable geometry and charge distribution, are recognized for their antiprotozoal potential, which can be caused most frequently through interactions with the parasite’s DNA, by enzyme inhibition, membrane association, by effects on transporters, or by host-mediated responses [67,68]. Based on this premise, Doganc et al. [38] performed an antiparasitic screening of a series of guanidinobenzimidazole derivatives, followed by in silico analyses to evaluate their interaction profiles with DNA. The stability of DNA–ligand complexes was further investigated through molecular dynamics simulations. In these studies, several pathogenic protozoa were tested for their in vitro susceptibility to the guanidinobenzimidazole derivatives, including *T. cruzi*.

**Figure 6:**
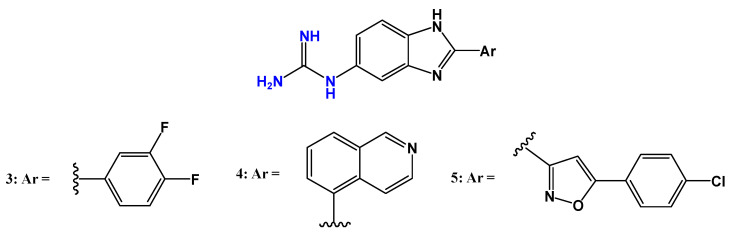
Guanidino-2-phenyl-1*H*-benzoimidazole derivatives with antitrypanosomal activity.

A series of guanidino-2-phenyl-1*H*-benzoimidazole derivatives was synthesized, and in vitro assays against the *T. cruzi* Tulahuen C4 strain were performed using benznidazole as the reference drug. None of the synthesized compounds showed greater potency than benznidazole (IC_50_ = 0.757 µg/mL), although compound **4** exhibited moderate activity (IC_50_ = 8.57 µg/mL) and low cytotoxicity toward rat skeletal myoblast L6 cells (CC_50_ = 44.6 µg/mL, Alamar Blue assay). Due to the limited biological activity observed, further in silico studies aimed at elucidating the mechanism of action against *T. cruzi* were not pursued by the authors. Additionally, in vitro assays against *T. b. rhodesiense* STIB900 revealed that compounds **3** (IC_50_ = 6.04 µg/mL), **4** (IC_50_ = 3.8 µg/mL), and **5** (IC_50_ = 5.62 µg/mL) (Figure 6) were the most active derivatives, although still significantly less potent than the reference drug melarsoprol (IC_50_ = 0.003 µg/mL). The relatively higher activity of compound **4** may be attributed to the presence of a nitrogen-containing aromatic heterocycle as a substituent, in contrast to most compounds in the series [38].

**Figure 7:**
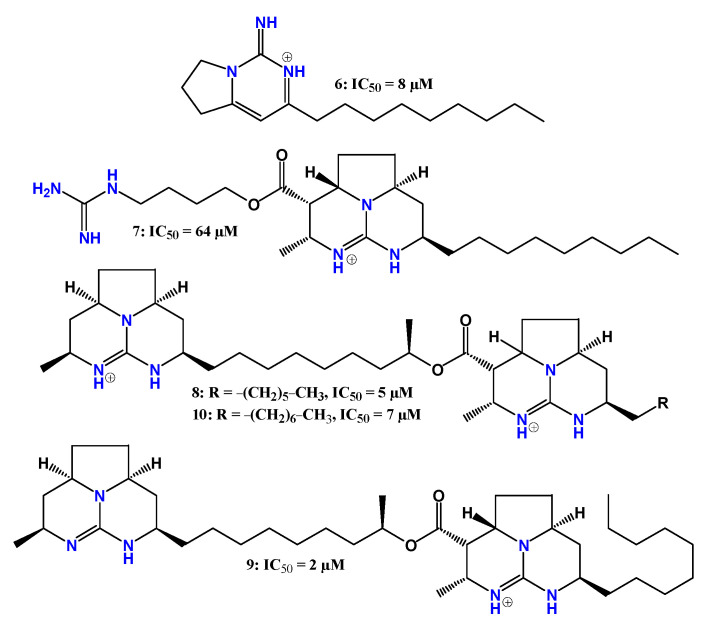
Guanidine alkaloids isolated from *Monanchora arbuscula*.

Natural guanidine alkaloids have also been investigated as potential antitrypanosomal agents. Santos et al. [69] evaluated guanidine-containing alkaloids isolated from the marine sponge *Monanchora arbuscula* (Figure 7). These compounds were obtained from a methanolic extract of the sponge and tested in vitro against trypomastigote forms of *T. cruzi*.

Although compounds **6** and **7–10** displayed higher activity than the reference drug benznidazole (IC_50_ = 441 µM), structure–activity relationship analysis suggested that the presence of two tricyclic guanidine systems contributes to improved biological activity. In particular, enhanced potency was observed when larger aliphatic carbon chains were attached to one of these tricyclic systems at carbon 27, while only one of the cyclic systems contained a cationic guanidinium group (monocationic compound **9**). The IC_50_ values indicated that these derivatives were at least 54-fold more potent than benznidazole, with compounds **9** and **10** displaying the highest selectivity indices based on cytotoxicity assays in LLC-MK2 monkey kidney cells [69].

**Figure 8:**
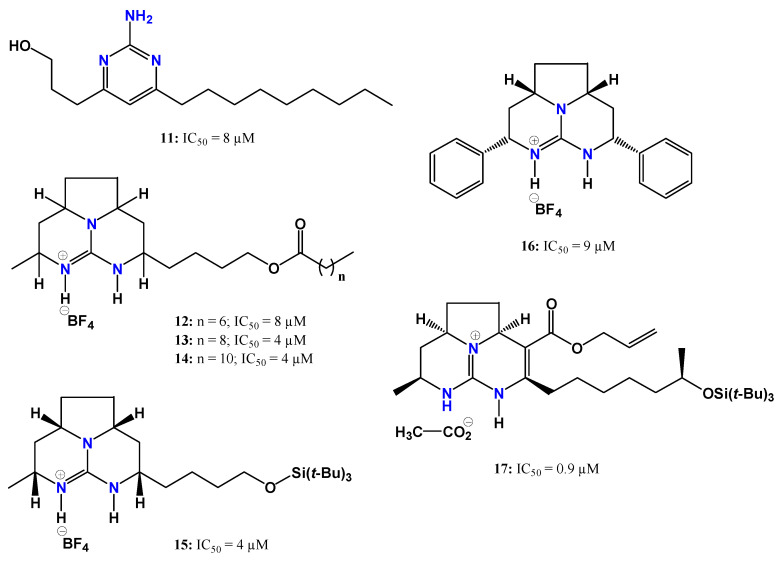
Chemical structures and biological evaluation of synthetic guanidine alkaloids.

Encouraged by the antitrypanosomal activity observed for natural guanidine alkaloids, Martins et al. [70] synthesized a series of 18 guanidine derivatives and evaluated their activity against trypomastigote forms of *T. cruzi*. The IC_50_ values ranged from 0.9 to 88.5 µM, with seven compounds showing higher potency than the reference drug benznidazole (IC_50_ = 16 µM) (Figure 8). Cytotoxicity was evaluated in mammalian NCTC (clone 929) cells.

Compound **17** exhibited the highest potency, being approximately 17-fold more active than benznidazole, although it also displayed high cytotoxicity (CC_50_ = 2 µM). Among the active derivatives, compounds **12–15** showed the most favorable toxicity profiles. Structure–activity relationship analysis indicated that the presence of a tricyclic guanidine system linked to an ester-containing side chain contributes to enhanced anti-*T. cruzi* activity, particularly in compounds with longer carbon chains attached to the ester carbonyl group (**13** and **14**). In compound **15**, the presence of an oxygenated silicon-containing moiety in this side chain also appeared to increase potency. Interestingly, a similar silicon-oxide group is present in the most potent derivative (**17**), although in this case, the presence of an allylic ester may have contributed to its increased toxicity [70].

During infection, the presence of *T. cruzi* triggers a cascade of immune responses in the host, many of which involve autoimmune and inflammatory processes that can severely affect cardiac muscle tissue [71]. At the same time, an effective inflammatory response is required for the control of parasitemia during the acute phase of infection [72]. Consequently, therapeutic strategies aimed at mitigating *T. cruzi* infection must achieve a balance between controlling parasite proliferation and preventing excessive immune-mediated tissue damage [73].

**Figure 9:**
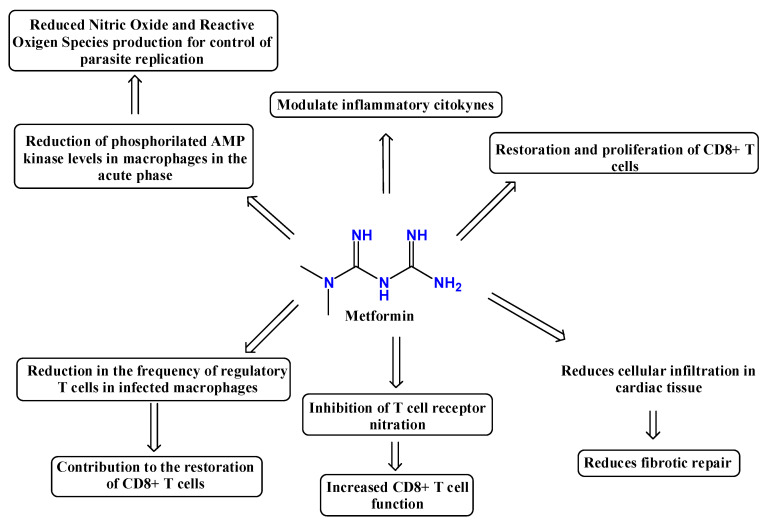
Proposed immunological and inflammatory effects associated with *T. cruzi* infection and metformin treatment in an experimental rat model.

The guanidine-containing antidiabetic drug metformin (Figure 9), known for its anti-inflammatory and immunomodulatory effects, was therefore evaluated by Baigorri et al. [72] for its impact on Chagas disease pathology in macrophages from rats infected with *T. cruzi*. The results suggest that metformin may modulate immune and inflammatory responses in infected peritoneal macrophages, leading to reduced parasitemia and protective effects on cardiac tissue in infected rats (these findings do not establish a clinically validated direct antiparasitic effect, and should be interpreted as model-dependent evidence). However, hepatic dysfunction was exacerbated by metformin treatment. This effect may explain the absence of significant differences in survival rates between treated and untreated infected animals [72].

**Figure 10:**
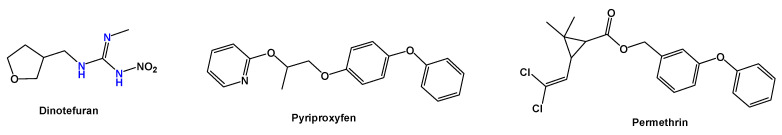
Components of the DPP tri-insecticide formulation.

Given the challenges associated with developing safe and effective drugs against *T. cruzi*, alternative strategies have also been explored to interrupt the parasite’s life cycle. In addition to difficulties in chemotherapy, increasing resistance of triatomine vectors to single-component insecticides has stimulated the evaluation of new vector control approaches. In this context, Tahir et al. [74] investigated the insecticidal efficacy of a topical formulation composed of dinotefuran, pyriproxyfen, and permethrin (DPP), in which dinotefuran is a neonicotinoid insecticide with a nitroguanidine moiety(Figure 10). The tri-insecticide formulation was administered at doses equivalent to the minimum recommended doses for dogs (64 mg/kg dinotefuran + 0.6 mg/kg pyriproxyfen + 46.6 mg/kg permethrin).

Topical application of the DPP formulation effectively prevented blood feeding by *Triatoma infestans* on treated rats. In untreated animals, feeding rates exceeded 94.3% throughout four weeks of observation, whereas in treated groups this value remained below 19.3% during the first three weeks, increasing to approximately 55% only after the fourth week. Moreover, insects feeding on treated animals exhibited strong toxic responses, with insecticidal efficacy exceeding 96% within 24 h of exposure during the first three weeks and high mortality rates throughout the study period. Comparative analyses with other insecticide classes suggested that the strong insecticidal effect of the formulation results primarily from the rapid activity of dinotefuran, which may have had its action potentiated by the presence of permethrin in the formulation.

These findings highlight the potential of guanidine-containing compounds not only as therapeutic agents but also as components of integrated strategies to control Chagas disease transmission. The application of such formulations in other important hosts within the parasite transmission cycle, such as dogs, may contribute to interrupting the epidemiological cycle of the disease.

### 2.2. Guanidine Derivatives Active Against Trypanosoma brucei

**Figure 11:**
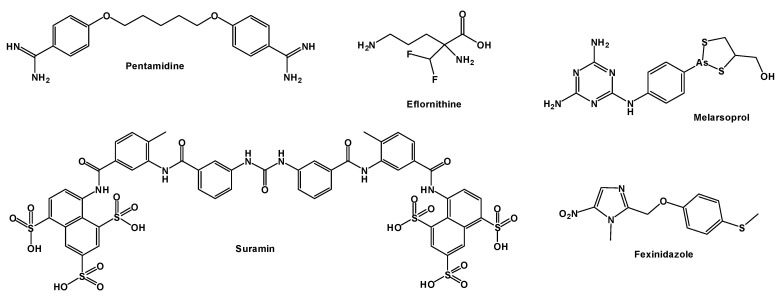
Commercial drugs used in the treatment of African trypanosomiasis.

Human African Trypanosomiasis (HAT), also known as sleeping sickness, is caused by a subspecies of *Trypanosoma brucei* and remains endemic in approximately 20 countries in sub-Saharan Africa. *T. b. gambiense* is responsible for most chronic infections in West and Central Africa, whereas *T. b. rhodesiense* causes acute disease in Eastern and Southern Africa [75,76]. Transmission occurs through the bite of infected tsetse flies and is associated with high morbidity and mortality [77,78]. Global health programs aim to eliminate the disease as a public health problem by 2030 [79]. Current treatment relies on drugs such as pentamidine, suramin, eflornithine, nifurtimox, fexinidazole, and melarsoprol (Figure 11). However, toxicity and emerging resistance limit their effectiveness [80,81]. Consequently, new chemotypes continue to be explored.

**Figure 12:**
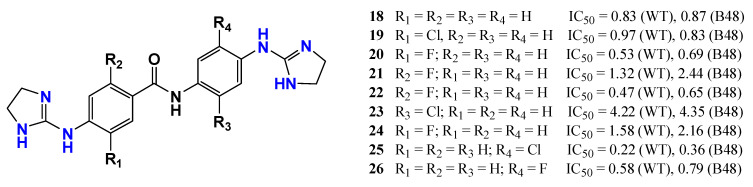
Dicationic guanidine derivatives synthesized by Ríos Martínez et al. [82].

Compounds bearing multiple guanidinium groups often display enhanced antitrypanosomal activity due to their dicationic character. Based on this knowledge, Ríos Martínez et al. [82] synthesized bis(2-aminoimidazoline) derivatives based on the lead compound **18** and investigated the influence of halogen substitution on the pKa values and on the biological activity. Fluorinated and chlorinated derivatives generally showed improved potency against both wild-type (WT) and resistant (B48) strains of *T. b. brucei*. For example, compounds **20**, **22**, and **26** displayed reduced IC_50_ values compared with the parent structure. The authors believe that the increased lipophilicity resulting from halogen substitution may also contribute to improved membrane permeation; however, experimental data were not presented in the study. It is important to point out that these effects may reflect a combination of inductive electronic modulation, changes in pKa, altered lipophilicity, conformational effects, binding-site complementarity, and, in some cases, possible halogen-bonding interactions. Notably, compound **25** exhibited superior activity against the resistant strain relative to pentamidine [82] (Figure 12).

**Figure 13:**
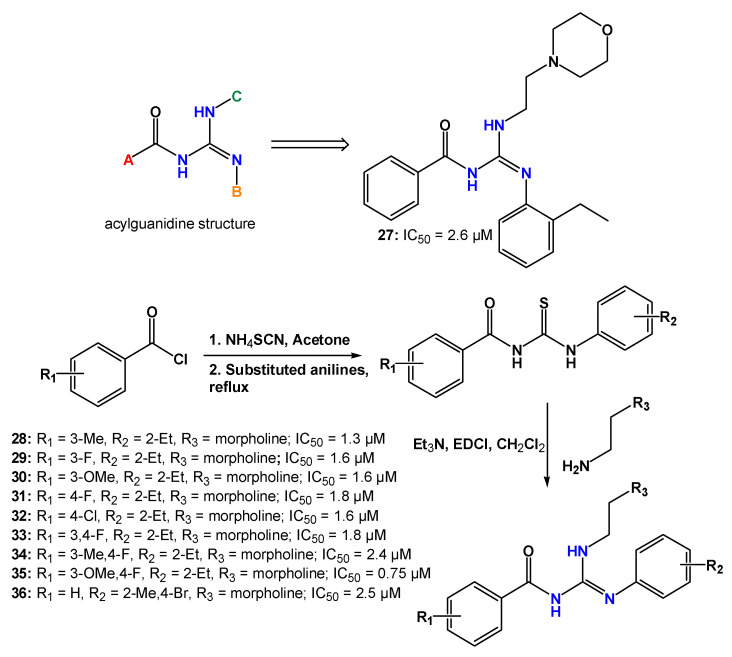
Chemical structures and biological results of guanidine derivatives **27–36** studied by Varghese et al. [83].

Further SAR insights were provided by Varghese et al. [83], who examined a series of acylguanidine derivatives in which substituent effects were explored across three structural regions. Substitutions at the *meta* and *para* positions of region A were generally well tolerated, particularly for electron-donating or halogen groups. In region B, a 2-ethyl aniline substituent proved optimal, whereas modifications to shorter or longer alkyl chains reduced potency. In region C, a morpholine ring connected through a two-carbon linker was essential for activity; increasing linker length or replacing morpholine diminished potency. The oxygen atom within morpholine likely contributes as a hydrogen-bond acceptor or participates in acid–base interactions with the molecular target. Figure 13 shows the chemical structure and the in vitro antitrypanosome results against strain 427 of *T. b. brucei* of the most promising compounds (compounds **28–36**).

**Figure 14:**
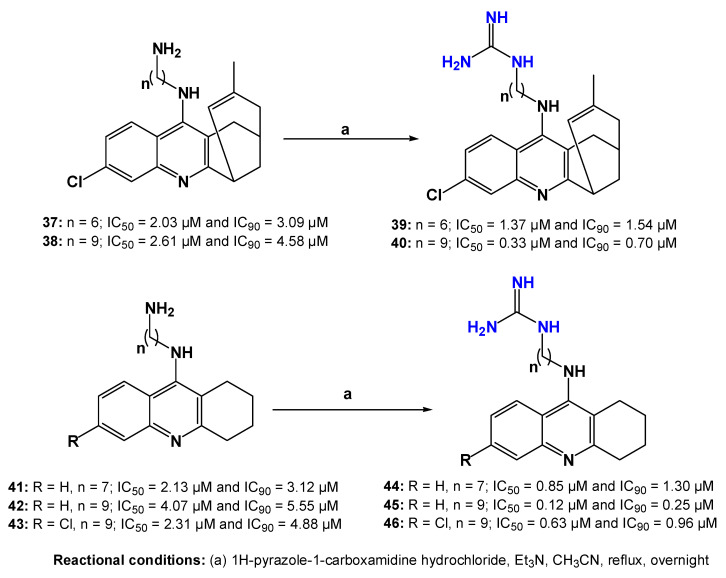
Huprino-guanidine and tacrino-guanidine derivatives with anti-*Trypanosoma* activity.

As previously mentioned, drug repurposing has emerged as a widely used strategy in the search for new antitrypanosomal agents, as it can significantly reduce development costs and shorten the time required for drug discovery [84]. At physiological pH, bisguanidines generally exist in a diprotonated form, a characteristic that may favor electrostatic interactions with biological targets and contribute to their antitrypanosomal activity [85,86]. Based on these considerations, Sola et al. [77] synthesized a series of guanidinylated huprine and tacrine derivatives derived from aminoalkyl-huprines and aminoalkyl-tacrines (**39**, **40**, **44**, **45**, and **46**—Figure 14), together with other nitrogen-containing analogs. The biological activity of these compounds was evaluated in vitro against bloodstream forms of *Trypanosoma brucei*, while their cytotoxicity was assessed in L6 mammalian cells.

Regarding the structure–activity relationship, the introduction of a guanidine moiety in compounds **39** and **40** significantly increased their antitrypanosomal activity compared with their corresponding amine precursors (**37** and **38**). Furthermore, elongation of the carbon spacer between the nitrogen atoms in compound **40** resulted in an additional improvement in biological activity. A similar trend was observed for compounds **44–46**, in which the presence of the guanidinyl group was also associated with enhanced antitrypanosomal potency. Overall, these guanidinylated derivatives exhibited higher activity than the reference drug nifurtimox (IC_50_ = 4.4 µM). However, most compounds showed moderate cytotoxicity toward L6 mammalian cells, with the exception of compound **44**, which displayed a more favorable toxicity profile.

**Figure 15:**
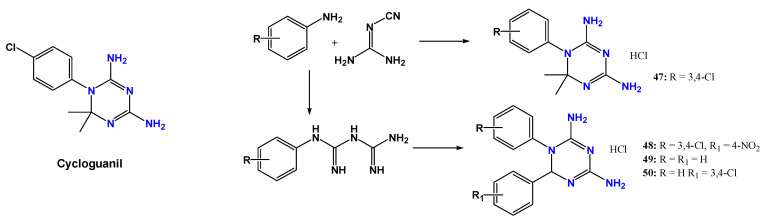
Cycloguanine-based guanidine derivatives are potential inhibitors of *T. brucei* pteridine reductase (*Tb*PTR).

Enzymatic targets within the folate pathway have also attracted attention. Pteridine reductase is an essential enzyme in trypanosomatids and represents a promising target for drug development [87,88]. Landi et al. [89] designed cycloguanine-derived inhibitors that interact with the enzyme’s substrate binding pocket. Compounds **47** and **48** (Figure 15) demonstrated high activity, with IC_50_ values of 0.692 µM and 0.186 µM, respectively. Docking studies indicated that halogen substituents contributed to interactions with residues such as Trp221, Leu209, and Ser207, while diguanidine groups stabilized hydrogen bonding within the active site. Despite promising enzymatic inhibition, both compounds exhibited cytotoxicity toward A549 cells (EC_50_ = 0.31 and 11.0 µM, respectively).

**Figure 16:**
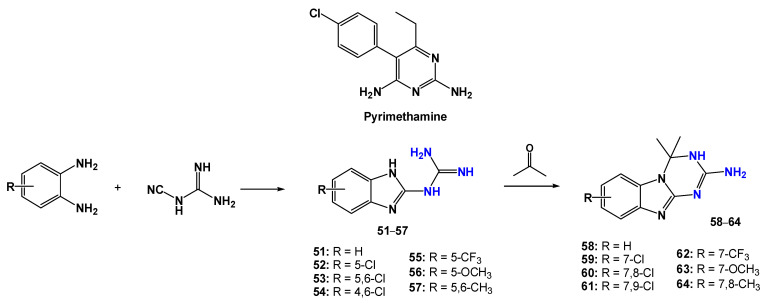
Bi− and tricyclic bisguanidine derivatives are potential inhibitors of *T. brucei* pteridine reductase (*Tb*PTR) and of dihydrofolate reductase (*Tb*DHFR).

Subsequent optimization efforts generated additional guanidine-containing scaffolds targeting both pteridine reductase (*Tb*PTR) and dihydrofolate reductase (*Tb*DHFR) [89]. Two series: 2-guanidinobenzimidazoles (**51–57**) and 2-aminotriazino [1,2-*a*]benzimidazoles (**58–64**) were synthesized and evaluated (Figure 16). The more flexible series (**51–57**) displayed stronger enzymatic inhibition, particularly against *Tb*DHFR. Compound **57** showed a Ki of 9 nM and high selectivity relative to the human isoform, while also inhibiting *T. cruzi* DHFR. In cellular assays, derivatives bearing lipophilic substituents such as Cl, CF_3,_ or CH_3_ exhibited the highest activity, with compounds **54** (IC_50_ = 2.91 µM) and **53** (IC_50_ = 7.8 µM) outperforming reference antifolates such as pyrimethamine and cycloguanil.

Subsequent optimization efforts led to the development of additional guanidine-containing scaffolds targeting both pteridine reductase (*Tb*PTR) and dihydrofolate reductase (*Tb*DHFR) [89]. Two series were synthesized and evaluated: 2-guanidinobenzimidazoles (**51–57**) and 2-aminotriazino[1,2-*a*]benzimidazoles (**58–64**) (Figure 16). The more flexible series **51–57** displayed stronger enzymatic inhibition, particularly against *Tb*DHFR. Among them, compound **57** showed a Ki value of 9 nM and high selectivity over the human isoform, while also inhibiting *T. cruzi* DHFR. Docking analyses indicated that these compounds bind to *Tb*PTR by occupying the active site of all subunits of the protein tetramer. The guanidino–benzimidazole moiety is positioned within the biopterin-binding region, allowing a π–π interaction with Phe97, while nitrogen atoms form hydrogen bonds with Tyr174, Ser95, and the NADP(H) cofactor, which act as key anchoring interactions. Substituents on the aromatic ring are oriented toward a hydrophobic region of the active site; in compound **57**, the –CH_3_ groups likely interact with residues in this pocket. Although some conserved interactions with pyrimethamine were observed, the absence of halogen substituents prevents the formation of a halogen bond with Trp221.

In vitro biological assays revealed that, except for compound **60**, the derivatives were not cytotoxic toward the human THP-1 cell line. Overall, compounds from series **51–57** showed higher anti-*T. brucei* activity than those from series **58–64**, particularly when lipophilic substituents (Cl, CF_3_, or CH_3_) were present. The most active compounds were the dichlorinated derivatives **54** (IC_50_ = 2.91 µM) and **53** (IC_50_ = 7.8 µM), which were more potent than the antifolate drugs pyrimethamine, cycloguanil, and methotrexate, although still less active than pentamidine. In contrast, the absence of substituents (as observed in **51**) or the presence of a polar group (5-OCH_3_ in **56**) resulted in loss of activity. The partial discrepancy between enzymatic inhibition and cellular activity may reflect differences in cellular uptake, intracellular exposure, or incomplete engagement of both *Tb*PTR and *Tb*DHFR. Therefore, impaired dual inhibition of the folate pathway should be considered a plausible hypothesis rather than a demonstrated mechanism [89].

**Figure 17:**
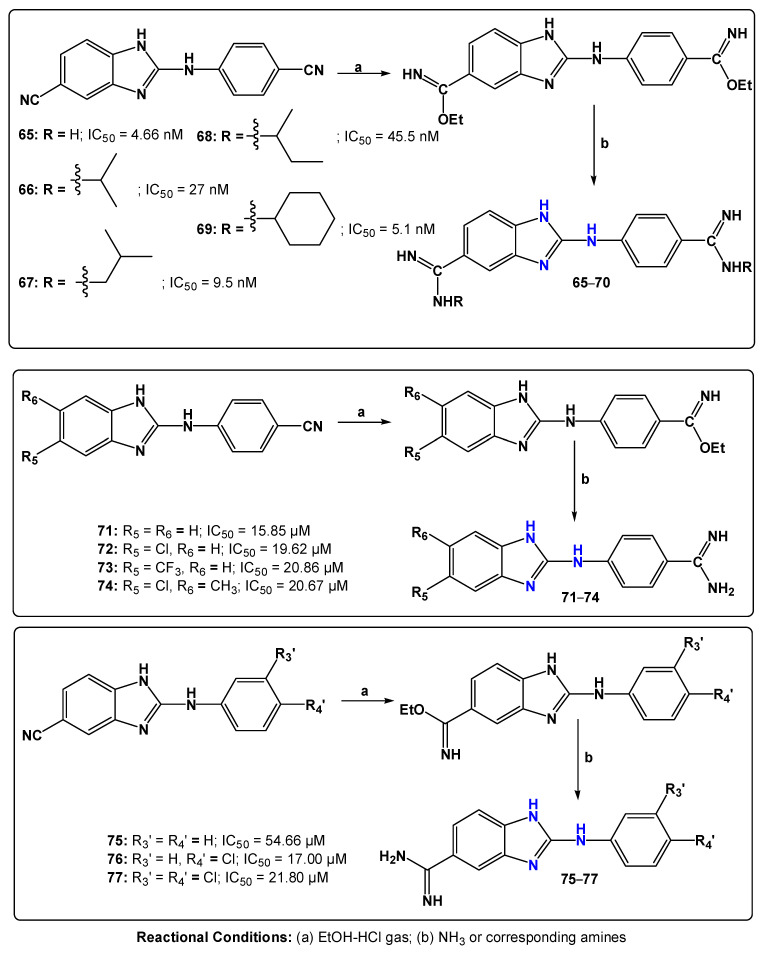
Mono/di amidino 2-anilinobenzimidazoles-based derivatives against *T. brucei rhodesiense*.

Karaaslan et al. [90] designed and developed a series of 2-anilinobenzimidazoles containing mono/di-amidino moieties capable of forming cationic groups, inspired by the chemical structure of the reference drug pentamidine (Figure 17). These compounds were evaluated for in vitro antiparasitic activity against bloodstream trypomastigote forms of *T. b. rhodesiense* STIB900, as well as for cytotoxicity in rat L6 myoblast cells.

The results highlighted the importance of the presence of two amidinium groups in the 2-anilinobenzimidazole core guanidinyl derivatives (compounds (**65–70**), which likely become dicationic at physiological pH. The loss of one of these amidinium groups, as observed for the mono-amidinium derivatives (**71–74** and **75–77**), resulted in a significant reduction in anti-*T. b. rhodesiense* potency. Among the dicationic compounds, **65**, **67**, and **69** were the most active, displaying IC_50_ values in the nanomolar range, with compounds **65** and **69** showing equipotency relative to the reference drug melarsoprol. The selectivity indices of the di-amidinium compounds were also markedly higher (SI ranging from 2060 to 22,600) compared with the mono-amidinium derivatives (SI ranging from 1.0 to 7.7).

**Figure 18:**
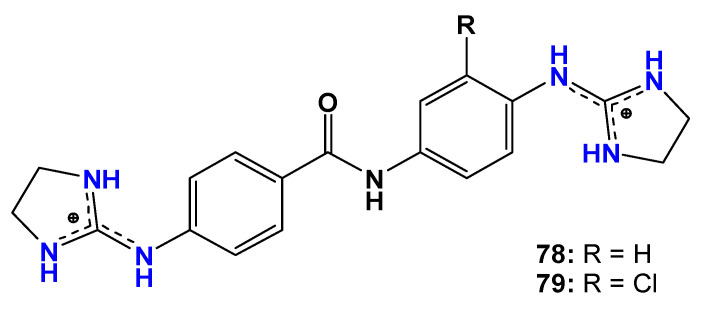
Chemical structures of *bis*(2-aminoimidazolium) compounds.

The dicationic profile of compounds is also considered important for anti-*T. brucei* activity in bis(2-aminoimidazoline) derivatives. Their positive charge allows high-affinity interactions with the minor groove of parasite DNA, particularly at adenine–thymine (AT)-rich sequences, thereby interfering with DNA-dependent enzymatic processes [91]. Based on this rationale, Millan et al. [92] investigated the affinity of two bis(2-aminoimidazolium) compounds (**78** and **79**, Figure 18) for AT-rich sequences of kinetoplast DNA, in addition to evaluating their antitrypanosomal activity against bloodstream forms of *T. brucei* in both in vitro assays and in vivo studies using the STIB900 mouse model of acute infection.

In vitro assays against *T. b. brucei* strain 427WT showed that compounds **78** (IC_50_ = 0.83 µM) and **79** (IC_50_ = 0.220 µM) were 52- and 14-fold less potent, respectively, than isometamidium (IC_50_ = 0.016 µM). The authors suggest that there is a possibility that these compounds exert their biological effects through binding to kinetoplast DNA, thereby interfering with the parasite cell cycle and inhibiting its growth and division. Transmission electron microscopy further revealed that the interaction of compounds **78** and **79** with kinetoplast DNA leads to its destruction after 24 h of exposure at a concentration of 5 µM [92].

In vivo studies demonstrated complete curative activity for compound **78** at doses of 4 × 5 mg/kg/day (i.p.) and 4 × 50 mg/kg/day (oral), and for compound **79** at 4 × 20 mg/kg/day (i.p.) in early stages of human African trypanosomiasis. The authors suggest that the antitrypanosomal activity of these compounds may be associated with their affinity for AT-rich regions of parasite DNA, as confirmed by surface plasmon resonance (SPR) biosensor experiments. Compound **79** was also shown to inhibit the binding of high-mobility group proteins HMGA1a and HMGB1 to DNA, thereby disrupting chromatin organization and gene expression. Additionally, binding of other DNA-dependent proteins may be impaired by the formation of stable complexes between the dicationic compounds and the DNA double helix. Crystallization studies of the DNA–compound **78** complex demonstrated that the dicationic guanidinium nitrogens are essential for minor-groove binding through hydrogen-bond formation with adenine and thymine nucleotides. Computational modeling further indicated a similar interaction pattern for compound **79** [92].

#### Structure–Activity Relationship Considerations for Antitrypanosomal Guanidines

Analysis of the studies discussed above reveals consistent structure–activity relationship (SAR) trends for guanidine-containing compounds active against *Trypanosoma* species. Despite the structural diversity of the evaluated scaffolds, the guanidine moiety emerges as a key pharmacophoric element contributing to antitrypanosomal activity.

Overall, cationic character plays an important role; mono- and, particularly, poly-guanidinium systems enhance biological activity by promoting electrostatic interactions with negatively charged biological targets, including enzyme active sites and parasite DNA. This behavior is especially evident for dicationic and bisguanidine derivatives, which often display improved potency and, in some cases, activity against resistant strains. In addition, substituent electronic effects significantly influence activity. The incorporation of electron-withdrawing groups, especially halogens such as fluorine and chlorine, frequently leads to enhanced potency. This effect is likely associated with both electronic modulation and increased lipophilicity, which may improve membrane permeability and interactions within hydrophobic regions of target proteins.

Molecular architecture is another critical parameter. The spatial arrangement between guanidine groups and aromatic or heterocyclic scaffolds, as well as the length and flexibility of linker units, strongly affects biological activity. Optimal spacer lengths enable appropriate positioning of cationic centers for interaction with key residues, whereas excessive rigidity or flexibility may reduce binding efficiency. Furthermore, hydrogen-bonding capacity is a key determinant of target affinity. The guanidine group acts as a strong hydrogen-bond donor and is consistently involved in stabilizing interactions within enzyme active sites and nucleic acid grooves, contributing to ligand recognition and binding stabilization.

Finally, pharmacokinetic limitations remain a major challenge. Although the high polarity and basicity of guanidinium groups favor target interactions, they may impair membrane permeability and oral bioavailability. For late-stage human African trypanosomiasis, central nervous system exposure would also need to be demonstrated experimentally, particularly because highly cationic compounds are not generally expected to cross the blood–brain barrier efficiently.

Taken together, these studies indicate preliminary and scaffold-dependent SAR tendencies for guanidine-containing and guanidine-related compounds with antitrypanosomal activity. Cationic charge distribution, electronic modulation, molecular architecture and geometry, hydrogen-bonding capacity, and lipophilicity, alongside critical pharmacokinetic constraints, may contribute to activity, but the available data remain insufficient to establish universal SAR rules across structurally diverse compound classes. And strategic optimization of these parameters is essential for the successful development of clinically viable antitrypanosomal agents.

### 2.3. Guanidine-Containing Compounds with Antileishmanial Activity

Leishmaniases comprise a group of parasitic diseases caused by different species of *Leishmania*. According to the Pan American Health Organization [93], epidemiological data from the Americas indicate a decrease in the number of cases from 67,949 in 2005 to 34,954 in 2023. This reduction has been observed in most countries, except for Argentina, Costa Rica, Ecuador, Guyana, Mexico, and Suriname, where the number of cases increased in 2023. However, despite this overall decline, there is growing concern regarding the increase in *Leishmania infantum*, the agent that causes visceral leishmaniasis, in countries across the Americas and in border regions of Southern Cone countries. Therefore, leishmaniases remain a public health concern, and considering that current treatment relies mainly on the administration of pentavalent antimonials and amphotericin B, both associated with low efficacy and significant toxicity, the development of new therapeutic alternatives remains necessary [94].

Guanidine-containing molecules have emerged as relevant scaffolds in medicinal chemistry programs targeting neglected diseases, including leishmaniasis. Over the last decade, multiple studies have investigated the antileishmanial potential of these compounds, revealing a diversity of structural classes and mechanisms of action.

**Figure 19:**
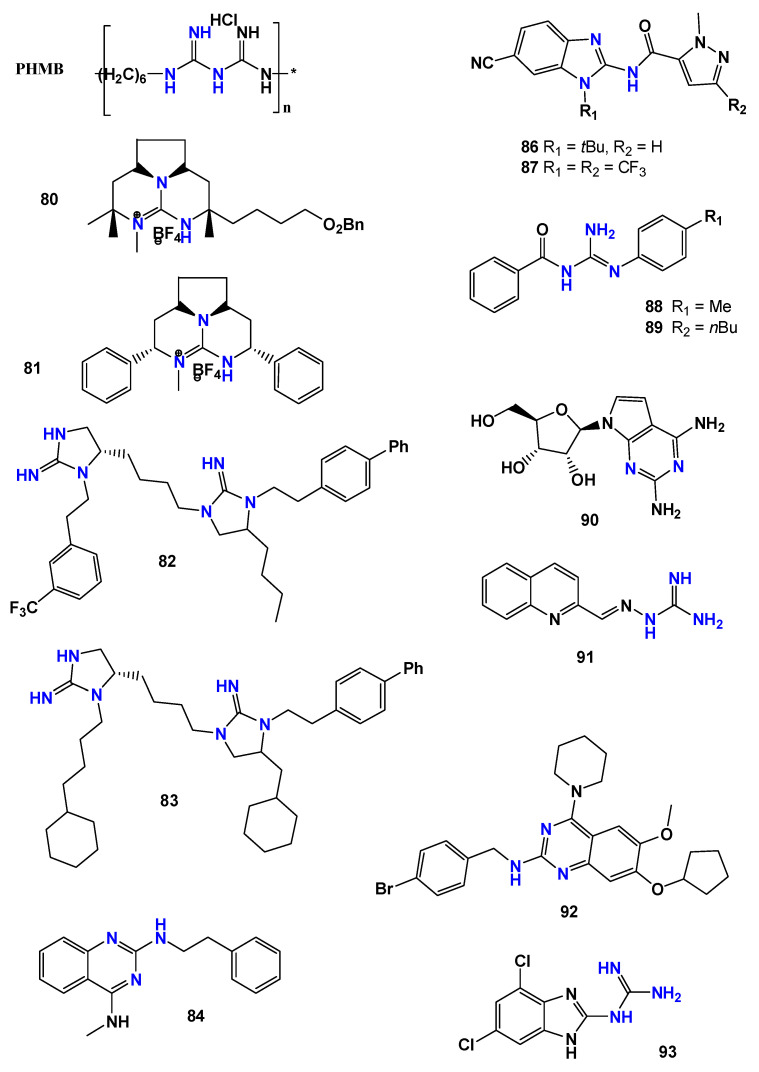
Guanidine derivatives with antileishmanial activity. * indicates that the structure is polymeric, and that the units between brackets are repeated.

Firdessa et al. [95] evaluated the antileishmanial activity of the antiseptic polyhexamethylene biguanide (**PHMB**) (Figure 19) against *Leishmania major*. The compound displayed IC_50_ values of 0.41 µM for promastigotes and 4.0 µM for amastigotes. However, despite this apparent potency, **PHMB** showed poor selectivity, with a CC_50_ of 4 µM in bone marrow-derived macrophages and an SI close to 1, indicating that it should be regarded as a high-potency but poorly selective hit rather than as a directly suitable antileishmanial lead.

Martins et al. [70] investigated synthetic analogs of marine guanidine alkaloids against *Leishmania infantum*. The compounds **80** and **81** (Figure 19) showed the most promising activity against intracellular amastigotes, with IC_50_ values of 5 µM and 2 µM, respectively, and selectivity indices of 25 and 20. In addition, these molecules were associated with the modulation of the anti-inflammatory response in infected macrophages.

Subsequent efforts focused on the identification of optimized guanidine scaffolds through library-based screening approaches. Using mixture-based combinatorial libraries, Giulianotti et al. [96] identified bicyclic guanidines as promising antileishmanial chemotypes. Structural refinement led to a series of 27 analogs, among which compounds **82** and **83** (Figure 19) exhibited potent activity against *Leishmania donovani* promastigotes (IC_50_ = 0.3 µM) with high selectivity indices (89 and 69, respectively).

A complementary strategy based on high-throughput screening was later described by Corman et al. [97]. Through a target-free fluorometric platform combined with combinatorial chemistry, 2,4-diaminoquinazoline derivatives were identified as potential leads. Among these compounds, molecule **84** (Figure 19) demonstrated strong activity against *L. donovani* amastigotes (IC_50_ = 0.37 µM), being more active than the reference drug miltefosine.

**Figure 20:**
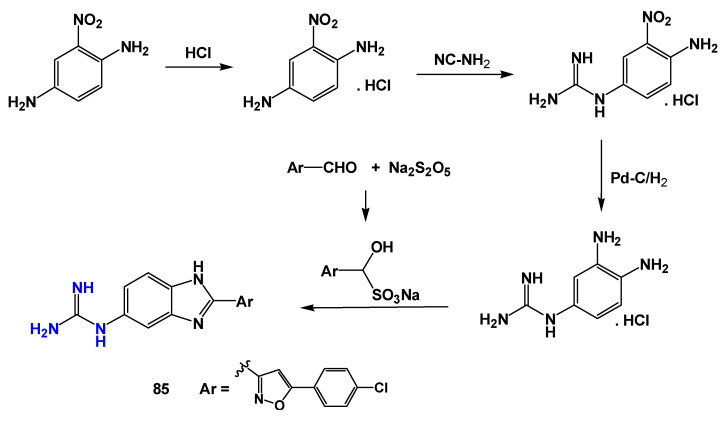
Synthesis of guanidinobenzimidazoles, including compound **85** described by Doganc et al. [38].

Further diversification of guanidine-containing scaffolds was achieved through the development of guanidinobenzimidazole derivatives. Doganc et al. [38] synthesized a series of monocationic analogs starting from 2-nitro-1,4-phenylenediamine through a four-step synthetic route (Figure 20). The compounds were evaluated against several protozoan parasites, including *Plasmodium falciparum*, *Trypanosoma brucei rhodesiense*, *Trypanosoma cruzi*, and *Leishmania donovani*. Although limited activity was observed against *T. cruzi* and *L. donovani*, the most active compound (**85**—Figure 20) showed an IC_50_ of 7.7 µg/mL against *L. donovani* amastigotes.

Additional optimization of benzimidazole-based derivatives was reported by Ferreira et al. [98], who investigated a series of 2-aminobenzimidazoles against *Leishmania infantum*. After initial in vitro screening, the most promising candidates were compounds **86** and **87** (Figure 19) with IC_50_ values of 4.10 µM and 0.53 µM, respectively, against amastigotes.

Continued exploration of guanidine-based structures led to the evaluation of benzoylguanidine derivatives. Santiago-Silva et al. [99] synthesized nine *N*^1^*,N*^2^-disubstituted, benzoylguanidines similarly to those obtained by do Espírito Santo et al. [100] and assessed their activity against *Leishmania amazonensis*. Compounds **88** and **89** (Figure 19) exhibited IC_50_ values of 90.8 µM and 68.4 µM against promastigotes and selectivity indices of 5.5 and 12.5, respectively. Although less potent than amphotericin B, these molecules reduced macrophage infection rates and presented favorable in silico ADME-Tox predictions, suggesting potential for further structural optimization.

Other studies have incorporated the guanidine functionality into distinct pharmacophoric frameworks. Lin et al. [101] described *N*6-modified 7-deazapurine nucleoside analogs, among which compound **90** (Figure 19) showed activity against *Leishmania infantum* intracellular amastigotes with an EC_50_ of 21.4 µM and a selectivity index greater than 2.99.

Similarly, aminoguanidine-derived hydrazones were investigated by de Aquino et al. [102] against *Leishmania chagasi* amastigotes. Within this series, compound **91** (Figure 19) showed the highest potency, presenting an IC_50_ value of 0.6 µM in amastigotes and a selectivity index of 52.5.

Further structural optimization of guanidine-containing scaffolds was described by Das et al. [103], who investigated 2-aminoquinazoline derivatives against *Leishmania donovani*. Among the evaluated compounds, **92** (Figure 19) displayed the highest activity, with an IC_50_ of 2.57 µM against promastigotes. Comparative analysis indicated that the presence of a guanidine moiety enhanced the antileishmanial activity.

Additional scaffold exploration was reported by Francesconi et al. [88], who evaluated amino-dihydrotriazine derivatives against *Leishmania infantum*. Among the evaluated molecules, compound **93** (Figure 19) exhibited the best activity, with an IC_50_ value of 6.62 µM against *L. infantum* promastigotes.

**Figure 21:**
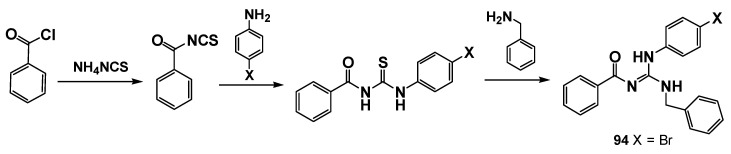
Synthesis of guanidine derivatives according to Espírito Santo et al. [100].

**Figure 22:**
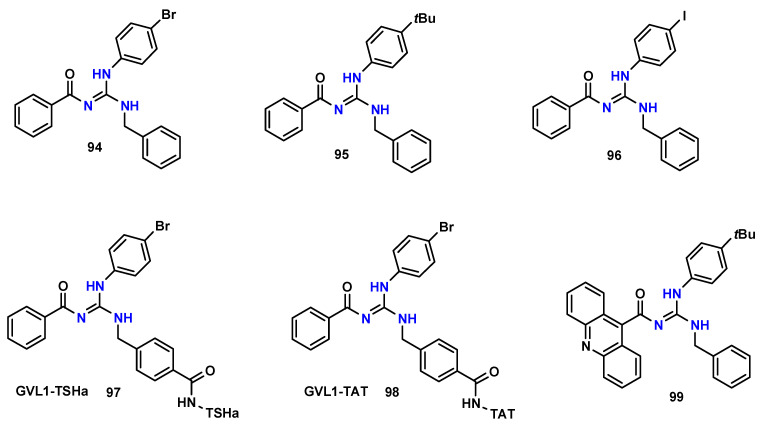
Guanidine derivatives and their bioconjugates with antileishmanial activity were synthesized by our group.

Our group also makes extensive contributions to the drug design and discovery process of new guanidine derivatives as potential antileishmania agents. In one of our first publications, and in parallel with screening-based discovery approaches, Do Espírito Santo et al. [101] reported the synthesis of ten trisubstituted guanidines. The synthesis was carried out in two steps, first through the obtention of thiourea intermediates by the reaction of benzoyl thiocyanates with anilines, followed by guanidine formation, reacting the thioureas with benzylamine through Bi(NO_3_)_3_·5H_2_O catalysis (Figure 21). Among the synthesized compounds, **94** (Figure 22) displayed the most promising profile against *Leishmania amazonensis* amastigotes, with an IC_50_ of 5.6 µM and a selectivity index of 131.8. Subsequent in vivo experiments in BALB/c mice revealed a 60% reduction in parasite burden at a dose of 0.25 mg/kg/day.

Beyond direct antiparasitic activity, guanidine derivatives have also been explored as inhibitors of parasite virulence factors. In this context, Moreira et al. [104] investigated the inhibitory activity of some tri-substituted guanidines published by do Espirito Santo et al. (2019) and demonstrated that some of them can inhibit the cysteine protease LmCPB2.8ΔCTE from *Leishmania major*. Among the evaluated molecules, **95** (Figure 22) inhibited approximately 73% of enzyme activity, with an IC_50_-CPB of 6.0 µM, while showing no activity against mammalian cathepsins B and L and no detectable hepatotoxicity or nephrotoxicity in vivo.

Continuing the investigation of the biological potential of trisubstituted guanidines published by do Espirito Santo et al. [100], Almeida et al. [105] evaluated the antileishmanial activity of compounds **94**, **95**, and **96** (Figure 22) against *Leishmania infantum*. These compounds displayed IC_50_ values against promastigotes of 12.7, 24.4, and 23.6 µM and against amastigotes of 26.1, 21.1, and 18.6 µM, respectively. Notably, **96** increased nitrite production in peripheral blood mononuclear cells, suggesting a potential immunomodulatory component.

Additional evaluation of trisubstituted guanidines against *Leishmania (Viannia) braziliensis* further supported their therapeutic potential. Dos Anjos et al. [106] reported IC_50_ values of 5.4 µM and 5.9 µM against promastigotes and 5.0 µM and 4.3 µM against amastigotes for **94** and **95** (Figure 22), respectively. Importantly, no cytotoxicity was observed in human cell lines (HEK-293, CaCo-2, and A549) at concentrations above 500 µM.

Conjugation strategies have also been explored by our group in order to enhance cellular uptake and biological activity. Costa et al. [107] modified compound **94** to enable conjugation with antimicrobial peptides, generating guanidine–peptide bioconjugates. A carboxylic acid group was incorporated into the para portion of benzylamine, subsequently allowing the production of two bioconjugates, GVL1-TSHa and GVL1-(p-Bt), reacting with the *N*-terminal end of the antimicrobial peptides TSHa (FLSGIVGMLGKLF) and p-Bt [(KKYRYHLKPF)_2_K-NH_2_]. Among these, **GVL1-TSHa** (**97**) (Figure 22) demonstrated pronounced activity against *Leishmania amazonensis*, with IC_50_ values of 5.4 µM (promastigotes) and 0.33 µM (amastigotes) and a selectivity index of 6060. The activity against amastigotes was comparable to amphotericin B but with markedly higher selectivity.

More recently, using the same peptide–guanidine bioconjugation approach, De Souza et al. [108] reported the development of a new bioconjugate with TAT peptide (YGRKKRRQRRR), the **GVL1-TAT** (**98**) bioconjugate (Figure 22). Although the TAT peptide alone showed no activity against *L. amazonensis* or *L. infantum*, the conjugate exhibited high potency, showing IC_50_ values of 5.35 µM (promastigotes) and 0.80 µM (amastigotes) with SI > 1250 against *L. amazonensis*, whereas IC_50_ values of 1.20 µM and 3.80 µM with SI > 526 against *L. infantum*. These findings suggest that bioconjugation improves intracellular delivery of the guanidine pharmacophore.

Finally, in our most recent work, and using the molecular hybridization strategy, Dos Anjos et al. [109] developed acridine–guanidine hybrids by replacing the benzoyl moiety of previously reported trisubstituted guanidines with an acridine scaffold. This modification resulted in compounds with enhanced activity. The most active molecule, **99** (Figure 22), displayed an IC_50_ value of 0.53 µM against both axenic and intramacrophagic amastigotes of *Leishmania (Viannia) braziliensis*, with a selectivity index of 347, highlighting the potential of this hybrid strategy for antileishmanial drug development.

#### Structure–Activity Relationship Considerations for Antileishmanial Guanidines

Analysis of the reported studies reveals several recurring structure–activity relationship (SAR) trends associated with guanidine-containing antileishmanial compounds. Although the evaluated scaffolds differ substantially in size, polarity, and architecture, the guanidine functionality often contributes to biological activity by modulating charge distribution, hydrogen-bonding capacity, and target recognition. Nevertheless, activity is frequently determined by the complete molecular scaffold, including heterocyclic frameworks, peptide conjugates, nucleoside-like structures, acridine hybrids, and other pharmacophoric elements. One consistent observation is the importance of cationic character. Highly protonated guanidine or biguanide groups may promote, in select compounds, electrostatic interactions with negatively charged biological targets, including parasite membranes, phospholipids, and acidic residues in enzymatic binding sites. Early results obtained with polyhexamethylene biguanide support this hypothesis, as strong activity was observed despite limited selectivity [95]. These findings suggest that electrostatic interactions contribute significantly to the initial antiparasitic effect. However, it is important to highlight that this is not a universal mechanism, and other guanidine-containing antileishmanial scaffolds may act through enzyme inhibition, DNA-associated interactions, intracellular transport processes, immunomodulation, or scaffold-specific mechanisms.

Another relevant trend concerns structural rigidity and scaffold organization. The identification of bicyclic guanidines through mixture-based libraries demonstrated that conformational restriction may enhance potency and selectivity [96]. Similar observations arise from heterocyclic derivatives such as guanidinobenzimidazoles and 2-aminobenzimidazoles, in which the heteroaromatic framework likely contributes to improved molecular recognition and intracellular stability.

Substitution patterns around the guanidine core also appear to strongly influence activity. Trisubstituted guanidines reported in several studies demonstrated favorable potency and selectivity profiles [100,105,106]. These derivatives suggest that appropriate steric and electronic modulation of the guanidine substituents may improve interactions with parasite targets while reducing toxicity toward host cells.

In addition, hybridization with other pharmacophores has emerged as an effective strategy to enhance activity. For example, incorporation of guanidine motifs into quinazoline and nucleoside-like scaffolds produced compounds with measurable antileishmanial effects [97,101,103]. These findings indicate that guanidine functionality can be successfully integrated into structurally diverse frameworks without loss of activity.

Another important SAR insight arises from conjugation strategies designed to improve intracellular delivery. Guanidine–peptide bioconjugates displayed markedly enhanced selectivity and potency compared with the parent molecules [107,108]. This behavior suggests that cellular uptake and subcellular localization represent key determinants of efficacy for guanidine-based compounds.

More recently, molecular hybridization approaches have provided further evidence of the versatility of this scaffold. Replacement of aromatic fragments with extended heteroaromatic systems, such as acridine, significantly improved potency against *Leishmania (Viannia) braziliensis* [109]. Such results indicate that increasing π-surface interactions and potential DNA or enzyme binding may contribute to enhanced activity.

Taken together, the available data suggest that antileishmanial activity in guanidine-containing compounds is influenced by a combination of factors, including cationic charge distribution, scaffold rigidity, substituent pattern, and cellular delivery mechanisms.

### 2.4. Guanidine-Containing Compounds with Antitubercular Activity

Tuberculosis remains among the leading causes of mortality worldwide, and currently available treatments show limited efficacy against latent or persistent bacterial populations. Consequently, therapy requires prolonged multidrug regimens frequently associated with systemic toxicity, which contributes to poor adherence and treatment discontinuation. These factors have accelerated the emergence of multidrug-resistant (MDR-TB) and extensively drug-resistant (XDR-TB) strains [44]. In this context, guanidine-containing compounds have attracted considerable interest as scaffolds for the development of new antitubercular agents. Structure–activity relationship (SAR) analyses indicate that the intrinsic basicity of the guanidinium group allows protonation under physiological conditions, generating cationic centers capable of establishing electrostatic interactions with negatively charged components of bacterial membranes. In the context of *M. tuberculosis*, these interactions may contribute to cell-envelope association, uptake, or target engagement, but should not be interpreted as evidence of a generalized membrane-disruptive mechanism [110].

**Figure 23:**
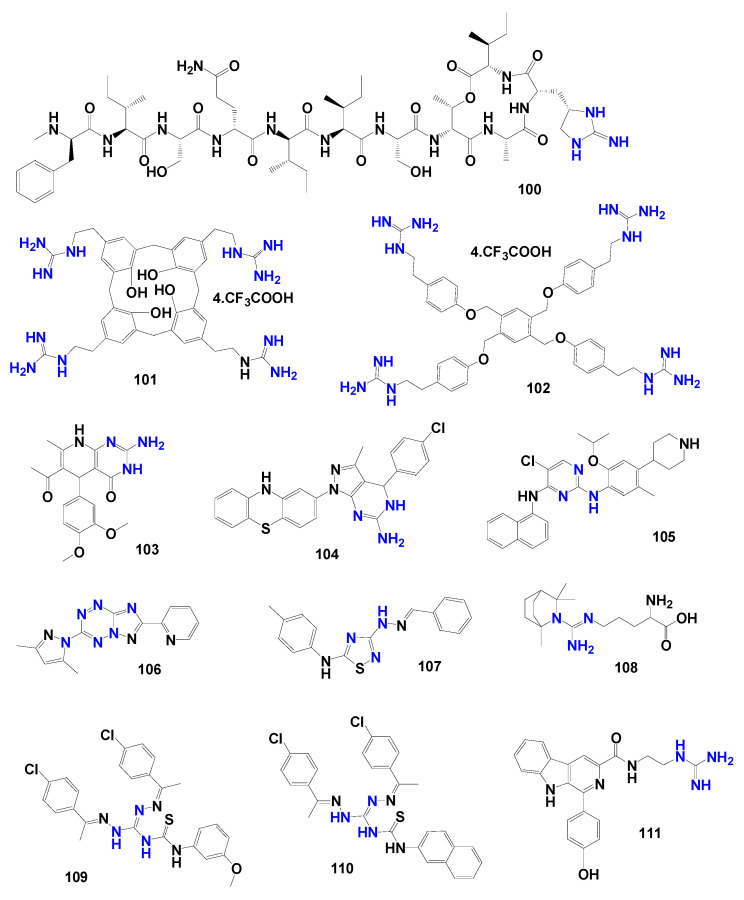
Guanidine derivatives with antitubercular activity.

Natural products further illustrate the pharmacological potential of guanidine-derived functionalities. As an example, the depsipeptide teixobactin **100** (Figure 23) showed a MIC of 0.125 μg/mL against *M. tuberculosis* H37Rv and a PD_50_ of 0.2 mg/kg in mice. Its antitubercular mechanism of action is associated with its binding to lipid II (peptidoglycan precursor, lipid III (teichoic acid precursor), and lipid pyrophosphate precursors of the bacterial cell wall through peptide backbone, including β-shett aggregation, and the unusual amino acid L-allo-enduracididine, a cyclic guanidine-containing residue. This interaction targets non-protein components of the cell wall biosynthetic machinery, thereby reducing the likelihood of resistance development. However, the structural complexity of teixobactin limits large-scale synthesis, and simplified analogs incorporating the same pharmacophoric motif have therefore been proposed as promising alternatives for drug design [111,112].

Beyond natural products, synthetic architectures incorporating multiple guanidinium groups have demonstrated notable activity against *Mycobacterium tuberculosis*. Preorganization of these functionalities within macrocyclic or planar frameworks appears to increase local cationic density and strengthen interactions with bacterial targets. For example, a tetra-*para*-guanidinocalixarene derivative **101** (Figure 23) adopts a three-dimensional cone conformation that facilitates membrane interaction and exhibits a MIC of 1 μg/mL against both the drug-sensitive H37Rv strain and the isoniazid-resistant MYC5165 strain of *M. tuberculosis*, with a selectivity index of 256 in human fibroblasts [113]. Compared to compound **101**, the tetra-guanidinium benzene derivative **102** (Figure 23) with a planar two-dimensional architecture produced an approximately eightfold increase in potency against resistant strains (MIC = 0.1 μM) together with reduced cytotoxicity (SI = 377). These findings suggest that modulation of conformational rigidity and spatial distribution of guanidinium groups may enhance antimycobacterial potency by altering cationic density, binding affinity, and permeability-related properties, rather than by acting on undefined mutated membrane targets [114].

The ability of guanidine moieties to mimic purines and essential cofactors such as NADH or folate also contributes to their biological relevance. When embedded within heterocyclic frameworks, including dihydropyrazolopyrimidines and dihydropyridopyrimidinones, these functionalities can occupy enzymatic active sites and enhance antimycobacterial activity. For instance, a 3,4-dimethoxyphenyl-substituted dihydropyridopyrimidinone derivative **103** (Figure 23) mimics key nitrogen-containing heterocycles involved in metabolic pathways, conferring selectivity toward slow-growing mycobacteria and enabling inhibition of efflux pumps associated with antimicrobial resistance. Nevertheless, additional structural optimization is required to improve solubility and potency against multidrug-resistant strains [44].

Similarly, the 4-chlorophenyl dihydropyrazolopyrimidine **104** (Figure 23) reported by Siddiqui et al. [115] exhibited low-micromolar activity (MIC = 0.02 μg/mL) against *M. tuberculosis* H37Rv, presenting a selectivity index greater than 500 in Vero cells, indicating that *para*- or *ortho*-substitution with strong electron-withdrawing groups may enhance biological performance within this class. In parallel, 2,4-diaminopyrimidine derivatives **105** (Figure 23) structurally related to ceritinib have demonstrated in vivo antimycobacterial activity (without exhibiting toxic effects until 300 mg/kg) through inhibition of dihydrofolate reductase (DHFR), a key enzyme in folate biosynthesis. This inhibition is a result of a broader interaction network involving hydrogen bonds with active-site residues, interactions with the NADPH-associated binding region, and aromatic contacts, which is also influenced by the interactions between the NH group of the piperidine substituent and the pyrimidine core within the enzyme active site [116].

Targeting enzymes involved in lipid biosynthesis has also emerged as a productive strategy for the development of guanidine-derived antimycobacterial agents. Enzymes such as β-ketoacyl-ACP synthase III (FabH) and enoyl-ACP reductase (InhA), both essential for the formation of long-chain fatty acids in the mycobacterial cell wall, represent validated molecular targets. Ishmetova et al. [117] demonstrated FabH inhibition by triazolotetrazines functionalized with pyridine amidines **106** (Figure 23), designed to mimic structural features of isoniazid [118]. Compound **106** showed an MIC value of 2.52 μg/mL against the *M. tuberculosis* H37Rv strain. In a related study, thiadiazolylhydrazone derivatives **107** (Figure 23) displayed activity against the H37Rv strain (31.54% inhibition at 50 μg/mL) while maintaining cellular safety in RAW 264.7 macrophages [118].

Complementary in silico investigations conducted by Sabarees et al. [119] suggested that active 1,8-cineole derivatives functionalized with *L*-arginine **108** (Figure 23) may use the de guanidinium group to engage hydrogen-bonding interactions within the NADH-dependent InhA active site, supporting activity against resistant strains. Compound **108** showed an MIC value of 1.6 µg/mL against *M. tuberculosis* H37Rv, and a binding affinity of –8.92 kcal/mol—PDB ID:2H9I [11].

Likewise, thiourea derivatives bearing aminoguanidine and bioactive thiadiazolylhydrazone fragments **109** and **110** (Figure 23) (MICs of 822.12 and 426.66 µM, respectively, against *M. tuberculosis* H37Rv) demonstrated that substitution with bulky hydrophobic rings, such as naphthyl groups, may enhance anchoring within the hydrophobic pocket of InhA, while the guanidinium moiety stabilizes the enzyme–ligand complex through cation–π or hydrogen-bonding interactions with Tyr158 (IFD score of −11.54 kcal/mol and −12.61 kcal/mol, respectively—PDB-ID: 4TZK) [110].

Conversely, β-carboline derivatives with a terminal guanidinio group **111** (Figure 23) had a reduced activity (MIC= 27.8 µg/mL against *M. tuberculosis* H37Rv), compared to other results mentioned, thus suggesting that condensed or heterocycle-integrated guanidine motifs may provide more favorable bioactivity profiles than flexible terminal groups [120].

#### Structure–Activity Relationship Considerations for Antitubercular Guanidines

Collectively, the available data reveal several consistent SAR patterns within guanidine-based antitubercular agents. First, protonatable guanidinium groups contribute to enhanced interactions with negatively charged membrane components and enzymatic residues, improving antimycobacterial potency. Second, spatial organization of multiple guanidine units, either in macrocyclic systems or planar aromatic frameworks, modulates cationic density and influences both activity and cytotoxicity.

Incorporation of guanidine motifs within heterocyclic scaffolds capable of mimicking purines or cofactors facilitates engagement with enzymatic targets such as DHFR, FabH, and InhA. Finally, hydrophobic substituents and electron-withdrawing aromatic groups frequently enhance binding affinity and biological activity, particularly against resistant strains.

### 2.5. Guanidine-Containing Compounds Investigated for Toxoplasma gondii

Current chemotherapy for toxoplasmosis relies primarily on the combination of pyrimethamine and sulfadiazine, which target two different enzymes within the folate biosynthetic pathway (dihydrofolate reductase (DHFR) and dihydropteroate synthase (DHPS)). Despite clinical utility, this regimen is associated with substantial toxicity, including bone marrow suppression, gastrointestinal disturbances, and systemic hypersensitivity reactions. In addition, these drugs act predominantly against tachyzoites, while latent bradyzoites within tissue cysts remain largely unaffected, enabling disease reactivation in immunocompromised individuals. Consequently, the identification of new chemical entities with improved selectivity and activity against multiple parasite stages remains a priority [121].

Within this context, guanidine-containing molecules have emerged as promising scaffolds for anti-*Toxoplasma gondii* drug discovery. Guanidine motifs are already present in several bioactive structures and frequently enhance interactions with biological targets through strong hydrogen bonding and electrostatic contacts.

**Figure 24:**
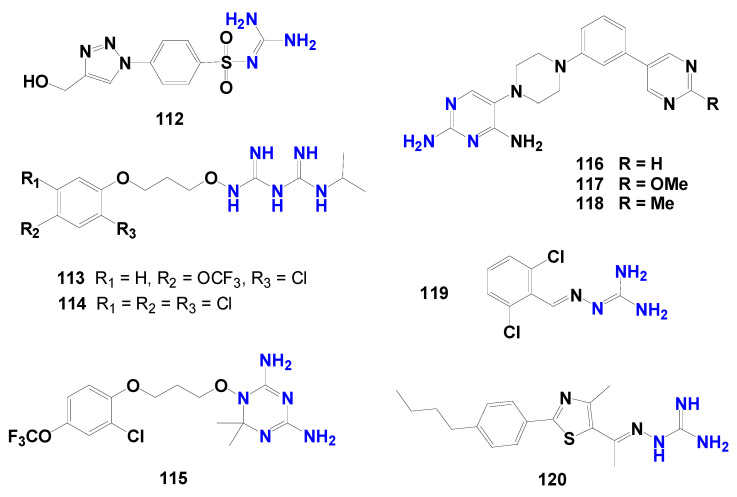
Guanidine derivatives with anti-*Toxoplasma gondii* activity.

A recent study by Arafa et al. [122] explored pharmacophore hybridization strategies derived from clinically used antifolates and demonstrated that incorporation of a guanidinium functionality was associated with improved antiparasitic activity relative to selected analogs containing only nitrogen heterocycles. The optimized compounds **112** (Figure 24) displayed nanomolar activity against tachyzoites (IC_50_ = 18 nM; SI = 29), highlighting the pharmacological relevance of this functional group.

Biguanide-based systems represent another important subclass. Some biguanides, such as **113** and **114** (Figure 24), can act as prodrugs that are metabolically converted into dihydrotriazine derivatives capable of inhibiting DHFR with nanomolar potency. The active metabolite **115** (Figure 24) inhibits *T. gondii* growth in culture (IC_50_ = 20 nM), exhibits strong enzymatic DHFR inhibition, with an IC_50_ value of 0.0065 μM against the target enzyme, and also reduces parasite burden in vivo at 1.25 mg/kg/day. The prodrug strategy increases aqueous solubility and enables oral administration, although some attenuation of biological potency has been reported, requiring higher dosing [45,46].

Further support for the relevance of guanidine-containing DHFR inhibitors was provided by Hopper et al. [123], who reported compounds with nanomolar potency and selectivity indices greater than 300. Structure–activity relationship analysis indicated that linking the 2,4-diamonopyrimidine moiety of pyrimethamine through the 5′-position to a 4-phenylpiperazine fragment enhanced selectivity toward *T. gondii* DHFR over the human enzyme [124]. The authors attributed these improvements to the favorable interactions pattern within the parasite DHFR active site, including hydrogen bonds with key residues interactions involving the NADPH-associated binding region, and optimized positioning of aromatic substituents within hydrophobic pockets. This design strategy enabled the development of three pyrimidine derivatives (compounds **116**, **117** and **118**) (Figure 24) exhibiting IC_50_ values below 5 nM, high selectivity, and pharmacokinetic properties compatible with oral drug development [123].

Drug repurposing has also been explored as an accelerated strategy for toxoplasmosis therapy. The guanabenz derivative (2,6-dichlorophenyl-*N*-guanidine) **119** (Figure 24) is notable for its ability to cross the blood–brain barrier and significantly reduce tissue cyst burden in the central nervous system. In murine models, treatment at 5 mg/kg once daily reduced cyst levels by approximately 69%, while doses up to 32 mg administered twice daily were considered safe. Mechanistically, guanabenz interferes with parasite translational control by inhibiting GADD34-mediated dephosphorylation of *T. gondii* eIF2α, thereby inducing cellular stress and impairing the viability of both tachyzoites and bradyzoites. However, clinical application is limited by its original pharmacological activity as an α_2_-adrenergic receptor agonist used for hypertension, which may cause severe hypotension in normotensive individuals. Consequently, the development of 2,6-dichlorophenyl-*N*-guanidine derivatives that retain eIF2α-mediated antiparasitic activity while minimizing adrenergic effects represents a rational direction for future research [121].

Additional nitrogen-rich scaffolds have also been described. For example, phenylthiazole derivative **120** (Figure 24) displayed activity in the micromolar range (IC_50_ = 2.1 μM) and showed a favorable safety profile, including cytotoxicity in Vero cells above 64 μg/mL and well tolerance in murine models (*Caenorhabditis elegans*) exposed to 40 μg/mL for four days [125,126]. These findings suggest a potentially less host-toxic mechanism of action and support continued exploration of structurally diverse guanidine-derived chemotypes.

#### Structure–Activity Relationship Considerations for Anti-*T. gondii* Guanidines

Collectively, the reported studies suggest several emerging SAR trends for guanidine-containing anti-*T. gondii* agents. First, incorporation of a guanidinium functionality appears to enhance target engagement and antiparasitic potency, likely due to strengthened electrostatic and hydrogen-bond interactions within enzyme active sites. Second, hybridization with antifolate-related scaffolds, particularly diaminopyrimidines, has been associated in selected studies with improved DHFR inhibition and parasite selectivity. Extension of the scaffold with hydrophobic substituents such as phenyl-piperazine groups can further increase affinity for the parasite enzyme relative to the human homolog. Finally, prodrug strategies and scaffold diversification may be useful approaches to modulate solubility, oral exposure, tissue distribution, and safety profiles, but these effects are compound-specific and require experimental pharmacokinetic and toxicological validation.

### 2.6. Compounds Bearing Guanidine Moieties Against Dengue

Dengue is a seasonal endemic disease that primarily affects tropical and subtropical regions. The World Health Organization estimates that approximately 400 million infections occur annually [127]. The etiological agent is the dengue virus (DENV), which comprises four serotypes (DEN-1–4) and is transmitted to humans by the female mosquito *Aedes aegypti* [128]. Clinical manifestations range from mild flu-like symptoms to severe forms such as dengue hemorrhagic fever and dengue shock syndrome, which may be fatal [129,130]. Although no specific antiviral therapy is currently available and treatment remains largely symptomatic, a prophylactic vaccine, Qdenga, produced by Takeda Pharmaceutical Company, has recently been introduced [131,132].

**Figure 25:**
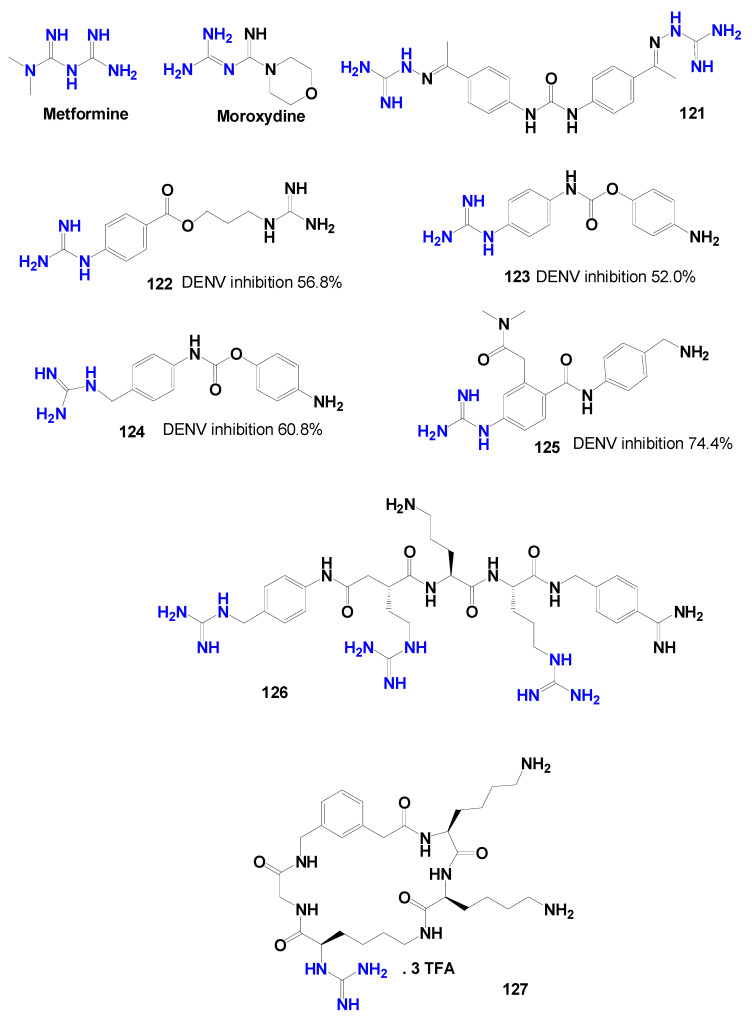
Guanidine-containing and guanidine-related compounds investigated against the dengue virus. Percentage inhibition values are shown with the corresponding test concentration when available and should be interpreted as screening data unless supported by IC_50_ or Ki determination.

Within this context, guanidine-related scaffolds, especially biguanides, have attracted attention due to their pleiotropic pharmacological properties. Metformin (Figure 25), a hypoglycemic drug belonging to the biguanide class, has been widely reported to exhibit anti-inflammatory, immunomodulatory, anticancer, cardioprotective, vasoprotective, and antimicrobial effects [133,134,135]. Clinical observations reported by Htun et al. [136] indicate that diabetic adults receiving metformin therapy present a reduced risk of developing severe dengue. Among 223 evaluated patients, 131 (58.7%) were treated with metformin and 92 (41.3%) were not. Metformin use was associated with an approximately 40% reduction in the risk of severe dengue according to the WHO criteria (aRR = 0.60; 95% CI: 0.37–0.98; *p* = 0.04), with a clear inverse dose–response relationship (aRR = 0.69; 95% CI: 0.49–0.98; *p* = 0.04). These findings suggest a potential protective role of metformin against disease progression in individuals with diabetes mellitus.

Complementary mechanistic insights were provided by Farfan-Morales et al. [137], who demonstrated that metformin exerts antiviral activity against several flaviviruses, including dengue virus. In cellular models, metformin inhibited infection by ZIKV, DENV, and YFV in a dose-dependent manner. However, the IC_50_ value reported for DENV was in the millimolar range (IC_50_ = 3.82 mM), indicating modest in vitro potency and suggesting that the observed effect is more consistent with host metabolic or immunomodulatory modulation than with direct classical antiviral activity. In vivo experiments further demonstrated that metformin produced a modest improvement in the course of DENV infection, increasing both median and mean survival from 13 to 15 days (*p* < 0.05) while attenuating severe clinical signs. These observations indicate that, despite structural similarities between flaviviruses, the antiviral response to metformin is virus-specific.

Another biguanide derivative investigated in the context of dengue is moroxydine (Figure 25), a heterocyclic biguanide characterized by the linkage of an external nitrogen atom to a diethylene-oxy bridge forming a morpholine ring. Although moroxydine has historically been reported to display broad antiviral activity, including limited reports involving dengue virus, it is not recognized as a clinically validated antiviral therapy for DENV.

Although moroxydine has historically been reported to display broad antiviral activity, including against adenovirus, influenza, herpesvirus, hepatitis viruses, paramyxoviruses, papillomaviruses, and dengue viruses [138], it is not recognized as a clinically validated antiviral therapy for DENV. Despite its low toxicity and broad antiviral activity, its clinical use gradually declined due to the absence of modern in vivo studies and randomized clinical trials. At present, moroxydine is mainly employed in veterinary medicine, although renewed interest has emerged due to activity against avian influenza and viral pathogens affecting aquatic organisms [139].

Beyond biguanides, small guanidine-containing molecules have also been explored as direct antiviral agents targeting dengue virus proteins. Naresh et al. [140] described the compound (2*E*, 2’E)-2,2’-(((carbonylbis(azanedidiyl))bis(4,1-phenylene))bis(ethan-1-yl-1-ylidene))bis(hydrazinecarboximidamide) **121** (Figure 25) as an inhibitor of dengue virus replication, displaying CC_50_ and IC_50_ of 13 and 500 μM, respectively. Despite demonstrating antiviral activity, its lack of selectivity (cytotoxicity exceeding antiviral activity) indicates that 121 is not a promising lead compound. Mechanistic studies indicated that **121** competes with the ligand *n*-octyl-β-*D*-glucoside (β-OG) within the hydrophobic pocket of the viral envelope protein E. STD-NMR experiments showed displacement of characteristic β-OG signals (0.8 and 1.3 ppm) in the presence of **121**, suggesting interaction with the hydrophobic pocket of the dengue virus envelope protein E. Structural analyses revealed intermolecular interactions with residues Gly200, Asp203, Gly271, Ser274, Thr48, Thr280, and Ala50, as well as hydrophobic contacts with Leu207. Although these data do not themselves establish a functional mechanism of viral entry or fusin inhibition, its identify **121** as a lead compound.

Targeting viral proteases has also proven to be a productive strategy for the development of guanidine-based antivirals. In the study conducted by Sundermann et al. [141], the authors synthesized a series of di-basic esters, amides, and carbamates using a dibasic 4-guanidinobenzoate derivative derived from camostat as a prototype. These compounds possess broad-spectrum serine protease and micromolar dengue protease inhibitory activity. Although some compounds, such as molecules **122–125**, showed inhibitory activity greater than 50% for DENV protease (Figure 25), these results were inferior to that of the prototype (DENV inhibition = 98.1%), demonstrating that the presence of the 4-guanidinobenzoate scaffold is essential for maintaining the anti-Dengue potential of these derivatives. However, because IC_50_ or Ki values were not determined for all compounds, percentage inhibition values were interpreted only as preliminary screening outcomes and were not used for direct potency ranking.

Consistent with these findings, Abdullah et al. [142] reported in their review several guanidinium-containing inhibitors of the dengue NS2B-NS3 protease. These compounds should be classified primarily as peptide-based or peptidomimetic competitive inhibitors, rather than as conventional drug-like small molecules, because their design derives from substrate-recognition elements of the protease.

Considering only the references published between 2015 and 2025, it was observed that in the work by Kouretova et al. [143], the authors described a peptide-based inhibitor of the dengue NS3 protease containing a guanidinium moiety. The guanidinium-peptide bioconjugate **126** (Figure 25), studied by the Kouretova group, a modified variant, 4-(guanidinomethyl)-phenylacetyl-Lys-Lys-Arg-NH2, was able to promote competitive inhibition of dengue virus protease (DENV2), exhibiting a Ki value at a low micromolar level (Ki = 1.22 μM).

Further structural refinement was achieved by Braun et al. [144], who developed macrocyclized substrate analogs incorporating strategically positioned guanidine groups at the P1 region to inhibit flaviviral NS2B-NS3 proteases, including those from Zika, West Nile, and dengue serotype 4. Structural studies demonstrated that conversion of the P1 amino group into a fully protonated guanidine, as observed in the compound, significantly improved anchoring within the S1 pocket through electrostatic interactions with Asp129. Comparison with non-guanidinylated analogs revealed more than a 300-fold decrease in Ki, highlighting the essential role of the guanidine moiety. Additional contacts with the carbonyl group of Gly159 and intramolecular stabilization of the macrocyclic scaffold further contributed to the subnanomolar affinity observed for derivatives such as compound **127** (Ki = 1.6 nM) (Figure 25). The marked decrease in Ki observed after guanidinium-containing modifications suggests a major contribution of the guanidine group to binding within this specific peptidomimetic series, although it does not establish an absolute requirement for guanidine across all dengue antiviral chemotypes.

#### Structure–Activity Relationship Considerations for Anti-Dengue Guanidines

Across the reported studies, several consistent SAR patterns can be identified. The presence of a protonated guanidinium group frequently contributes to potency in selected dengue-active scaffolds by supporting hydrogen-bonding and electrostatic contacts, but it should not be considered universally essential because activity remains highly scaffold- and target-dependent. The spatial positioning and accessibility of the guanidine moiety significantly influence inhibitory potency, particularly in protease inhibitors. Increased basicity and proper orientation of the guanidinium group enhance binding to catalytic pockets such as S1, while removal or substitution of this functionality results in substantial loss of activity. Nonetheless, target engagement by guanidinium-containing dengue inhibitors cannot be explained solely by electrostatic attraction. Binding also depends on pocket geometry, desolvation effects, specific hydrogen-bond networks, hydrophobic complementarity, and the spatial orientation imposed by the complete molecular scaffold. Finally, macrocyclization and scaffold rigidification appear to stabilize binding conformations and promote high affinity toward viral enzymes.

### 2.7. Guanidine-Containing Compounds with Antischistosomal Activity

Schistosomiasis is classified as an endemic disease by the WHO and affects millions of individuals living in regions with inadequate sanitation and limited access to clean water [145]. The disease is caused by the helminth *Schistosoma mansoni*, whose heteroxenous life cycle involves freshwater snails of the genus Biomphalaria as intermediate hosts and humans as definitive hosts. Acute infection may be present with fever, myalgia, and abdominal pain, whereas chronic disease is associated with hepatomegaly and rectal bleeding. Pharmacological treatment relies primarily on Praziquantel, which has remained the only widely validated therapy since 1973 [146,147]. Praziquantel belongs to the pyrazinoisoquinoline class and is considered a low-cost drug with well-established efficacy and safety. Nevertheless, it shows limited activity against immature parasite stages, and the potential emergence of resistance has stimulated efforts to identify alternative therapeutic strategies [148,149]. In this context, guanidine derivatives occupy a relevant position in nitrogen heterocycle chemistry due to their pronounced nucleophilicity and their capacity to generate functionalized polycyclic systems with diverse biological activities. Within drug discovery programs targeting schistosomiasis, the guanidine group may influence biological activity mainly through its high basicity, cationic character under physiological conditions, and ability to participate in hydrogen-bonding and electrostatic interactions with biological targets.

**Figure 26:**
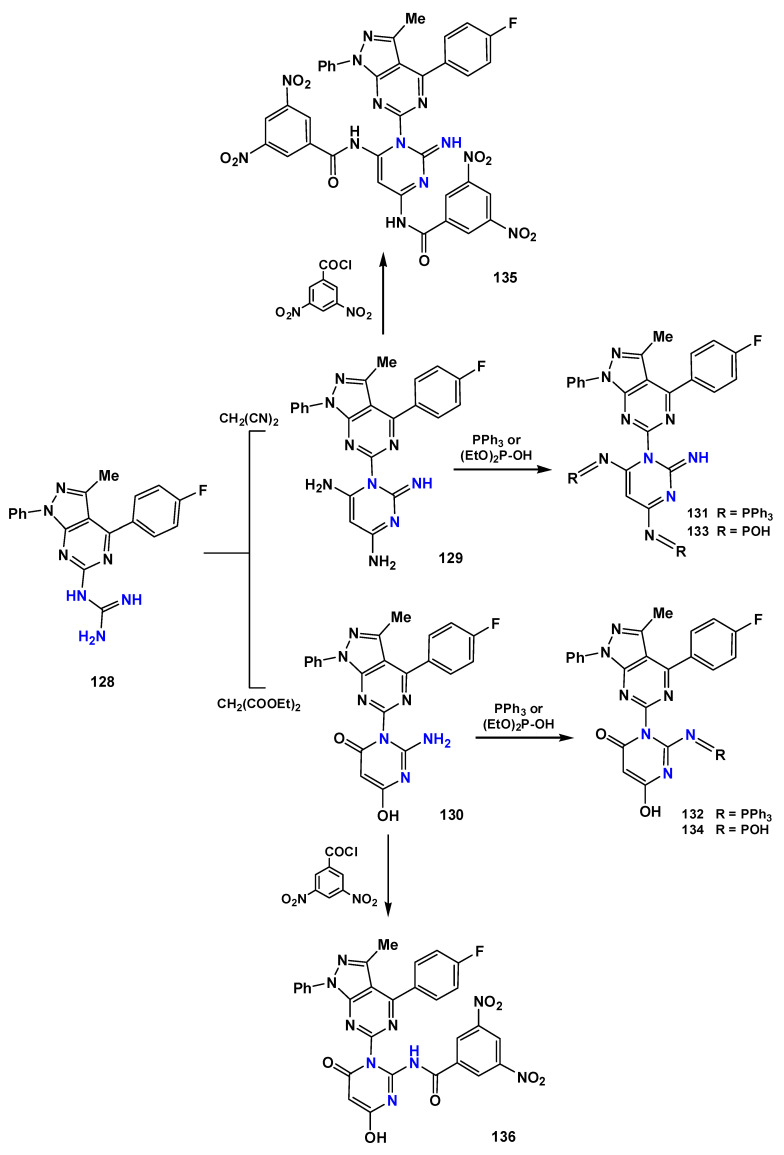
Synthesis of 1-heteroaryl guanidines (**129** and **30**), triphenylphosphine derivatives (**131** and **132**), hydroxyphosphinimino-pyrimidines derivatives (**133** and **134**), and aroyl amidopyrimidines derivatives (**135** and **136**) as molluscicidal agents.

An illustrative example was reported by Abed Bakhotmah [150], who employed guanidine as a key precursor in the synthesis of fluorinated pyrazolo[3,4-*d*]pyrimidines functionalized with phosphorus or nitrated aroyl groups (Figure 26). These compounds were evaluated against *Biomphalaria alexandrina*, the intermediate host of schistosomiasis, allowing systematic investigation of how structural variation influences molluscicidal activity. Biological evaluation revealed that compounds **129** and **130,** directly derived from 1-heteroaryl guanidine (**128**), showed low molluscicidal activity, producing only 20–30% mortality at 100 ppm.

In contrast, compounds in which the guanidine moiety participated in the formation of N=PPh_3_ or N=P–OH systems (compounds **131–134**) displayed enhanced efficacy, reaching 50–100% mortality at the same concentration. Notably, compound **133**, in which the guanidine core was converted to an N=P–OH functionality, exhibited the highest activity, producing 100% mortality at 100 ppm, 30% at 50 ppm, and 20% at 25 ppm, matching the inhibitory activity of the Baylucide (positive control). Derivatization of compounds **129** and **130** with 3,5-dinitrobenzamide substituents (compounds **135** and **136**) resulted in derivatives with moderate activity (50% and 30% mortality at 100 ppm, respectively). Collectively, these observations indicate that the presence of N=P-containing fragments may alter the electronic distribution of the scaffold, modulating the bioactivity.

**Figure 27:**
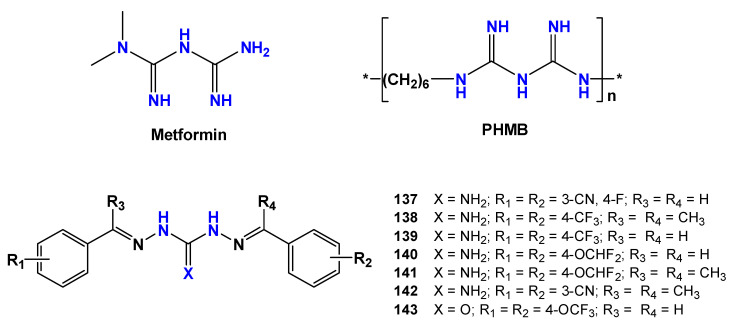
Guanidine derivatives with antischistosomal activity. * indicates that the structure is polymeric, and that the units between brackets are repeated.

Additional insights into guanidine-containing systems were provided by de Oliveira Melo et al. [151], who evaluated polyhexamethylene biguanide hydrochloride (**PHMB**) (Figure 27), a polymeric guanidine derivative previously reported to exhibit antimicrobial and antileishmanial activity. The study demonstrated that PHMB exerts toxicity against *Biomphalaria glabrata* across multiple developmental stages, with embryos showing the highest sensitivity. Control embryos exhibited low mortality (15 ± 5%) and high hatching rates (85 ± 5%), whereas PHMB reduced hatching to 9 ± 8% at 1.0 mg/L and 6 ± 5% at 1.6 mg/L, with complete inhibition (100%) at concentrations ≥2.0 mg/L. A concentration-dependent lethal toxicity profile was also observed, with LC_50_ values of 0.98 mg/L for embryos, 1.43 mg/L for newly hatched snails, and 1.49 mg/L for adults. Behavioral alterations associated with toxic stress were also observed, including shell retraction, lethargy, and excessive mucus secretion at concentrations as low as 1.6 mg/L. Although low LC_50_ values indicate measurable molluscicidal activity, these values should be interpreted cautiously and contextualized relative to standard molluscicides, environmental toxicity benchmarks, and non-target organism effects before any practical relevance can be inferred.

Host-directed therapeutic strategies involving guanidine-related compounds have also been explored. Salama et al. [152] evaluated the effects of metformin (MET) (Figure 27) in mice infected with *Schistosoma mansoni*. Treatment with praziquantel (PZQ) significantly reduced parasite burden, decreasing total worm numbers in 81.9%, whereas the combination PZQ/MET achieved an 83.5% reduction, comparable to praziquantel alone. Metformin alone did not significantly reduce worm burden but markedly decreased egg counts. In the liver, substantial reductions were observed with MET, PZQ, and PZQ/MET, while in the intestine, PZQ eliminated eggs completely and MET produced a pronounced reduction. The study concluded that although metformin does not directly affect adult worms, it significantly reduces egg production and attenuates inflammatory and fibrotic responses, particularly when combined with praziquantel. This effect should be interpreted primarily as a host-directed metabolic or immunomodulatory response rather than as evidence of direct antischistosomal activity against the parasite.

More direct antischistosomal activity has been reported for small guanidine derivatives. Lotz et al. [43] investigated compounds containing a symmetrical aminoguanidine core (Figure 27) and demonstrated that structural integrity of this motif is critical for activity within this scaffold series. Among the 80 compounds evaluated, 26 stood out, and 15 of them exhibited low EC_50_ values against both Newly Transformed Schistosomula (NTS) (1.12–4.63 μM) and adult *Schistosoma mansoni* worms (2.78–9.47 μM), indicating that subtle structural variations around the guanidine core significantly influence potency.

For the substituent groups on the phenyl rings (R_1_ and R_2_), it was observed that substitution by electron-withdrawing groups in the *para* position (e.g., –CF_3_, –OCF_3_, –F) is favorable for activity. Methyl substituents adjacent to the guanidine group (R_3_ and R_4_) markedly enhanced activity. For instance, compound **138** showed 68% inhibition in NTS, whereas the methylated analog **139** reached 96%, reducing the EC_50_ from 6.23 μM to 1.67 μM. A similar trend was observed for compounds **140** and **141**, with the methylated derivative **141** achieving 99% inhibition in adults and an EC_50_ of 2.78 μM compared with 64% inhibition and an EC_50_ of 5.12 μM for the non-methylated analog. In this series, the guanidine core also proved sensitive to modification of the imine atom: substitution with oxygen (in compound **143**) drastically reduced activity to 15%, demonstrating that the N–C(=NH)–NH arrangement is essential for the mechanism of action. Strongly electron-withdrawing substituents on the phenyl rings, including cyano, nitro, trifluoromethoxy, and difluoromethoxy groups, were associated with increased activity within this scaffold series. The presence of these electron-withdrawing substituents associated with the guanidine moiety modulates electronic distribution, acidity/basicity, lipophilicity, and binding-site complementarity, thereby influencing the biological activity. This effect was observed in compounds **137** and **142**, which achieved 100% inhibition in NTS and EC_50_ values of 1.25 μM and 1.12 μM, respectively. Despite the potent in vitro activity, the best compounds (**37** and **141**) failed to significantly reduce parasite burden in vivo, with reductions below 20%.

Although some derivatives displayed encouraging in vitro activity, their failure to reduce parasite burden by more than 20% in vivo indicates a major translational limitation and/or pharmacokinetic constraints associated with guanidine moieties, including high basicity, strong physiological ionization, low solubility, and potentially limited intestinal permeability. This discrepancy suggests that inadequate bioavailability, metabolic instability, limited tissue exposure, or insufficient parasite-site accumulation may compromise efficacy despite favorable in vitro profiles.

#### Structure–Activity Relationship Considerations for Anti-Schistosoma Guanidines

Taken together, the available evidence indicates that guanidine-containing compounds represent a versatile structural class for the development of antischistosomal and molluscicidal agents. Their activity spans multiple biological levels, ranging from direct toxicity against the intermediate snail host to modulation of parasite biology and host inflammatory responses. From a structure–activity perspective, several trends emerge. First, preservation of a guanidinium-like arrangement is often important in small-molecule series in which cationic and hydrogen-bonding interactions contribute to target recognition, but masked guanidines, prodrug forms, and bioisosteric replacements may also be viable depending on the scaffold and biological context. Second, electronic modulation of the guanidine group, either through interaction with phosphorus atoms or through electron-withdrawing substituents on adjacent aromatic rings, significantly enhances potency. Steric optimization around the guanidine core, such as methyl substitution, can substantially increase activity against both schistosomula and adult worms.

## 3. Materials and Methods

### 3.1. Search Strategy

A comprehensive literature search was conducted using two major scientific databases: PubMed and Web of Science. Because this review was designed as a narrative medicinal chemistry review rather than a systematic review, the search was complemented by manual screening of relevant references cited in the selected articles. The search was restricted to studies published between January 2015 and December 2025, in order to capture the most recent advances in the field of guanidine-based compounds applied to neglected diseases. The search strategy combined the term *“guanidine”* or *“guanidines”* with disease-specific keywords using Boolean operators. The following combinations were employed: (a) (*guanidines* AND (*leishmaniasis* OR *Leishmania*); (b) (*guanidines* AND (*Chagas disease* OR *Trypanosoma cruzi*); (c) (*guanidines* AND (*human African trypanosomiasis* OR *Trypanosoma brucei*); (d) (*guanidines* AND (*tuberculosis* OR *Mycobacterium tuberculosis*); (e) (*guanidines* AND (*toxoplasmosis* OR *Toxoplasma gondii*); (f) (*guanidines* AND (*dengue* OR *Aedes aegypti*); (g) (*guanidines* AND (*schistosomiasis* OR *Schistosoma*). Unlike other NTDs, the search for articles related to dengue also considered the vector-related term (*Aedes aegypti*). Only articles that specifically addressed insecticidal or vector control strategies were included.

This article is a narrative medicinal chemistry review and was not designed as a systematic review. Therefore, PRISMA-based article selection, quantitative screening metrics, and formal risk-of-bias assessment were not applied.

### 3.2. Eligibility Criteria

Studies were considered eligible if they met the following inclusion criteria: (a) reported at least a structurally identifiable guanidine, guanidinium, guanidino, guanidine-containing, or guanidine-related moieties that were relevant to the medicinal chemistry discussion, SAR interpretation, biological activity, or proposed mechanism of action. Compounds in which such motifs were merely peripheral and not chemically or pharmacologically discussed were not emphasized in the analysis; (b) presented experimental biological activity data (in vitro and/or in vivo) against pathogens associated with neglected tropical diseases (NTDs); (c) were original research articles, including studies involving synthetic compounds, natural products, or drug repurposing approaches; (d) were published in peer-reviewed journals within the defined time frame.

Dengue-related studies were included because dengue is an emerging and neglected arboviral disease of major relevance in tropical and subtropical regions, although it was analyzed separately from protozoan and helminthic NTDs.

Exclusion criteria included: (a) duplicate records; (b) review articles used as primary sources of biological activity; (c) patents without peer-reviewed experimental validation; (d), preprints not subjected to peer review; (e) studies lacking experimental biological data; (f) studies lacking chemical structures or sufficient biological data, and docking-only studies without experimental biological validation; (g) reports not clearly specifying the chemical structure or the presence of a guanidine functional group; (h) studies not directly related to the targeted NTD pathogens. Review articles were consulted only for contextual background or to identify original primary studies.

### 3.3. Study Selection and Data Synthesis

The selection process was performed through an initial screening of titles and abstracts, followed by full-text evaluation of potentially relevant articles. From the selected studies, relevant data were systematically extracted, including: chemical structure and classification of guanidine derivatives, synthetic or natural origin, target pathogen, type of biological assay, reported activity (e.g., IC_50_, EC_50_), selectivity indices when available, and proposed mechanisms of action.

In this review, IC_50_ values are used preferentially for enzyme or target inhibition assays when reported by the original authors, whereas EC_50_ values are used for cellular efficacy assays. When the original study used a different terminology, the original designation was preserved, and the assay context was specified to avoid ambiguity.

The level of evidence was explicitly distinguished throughout the review. Computational docking and molecular modeling results were interpreted as hypothesis-generating data; enzymatic inhibition values were treated separately from cellular IC_50_ or EC_50_ values; cytotoxicity data were reported with the corresponding mammalian model; and in vivo efficacy was discussed only when animal model data were available.

The collected data were qualitatively analyzed with a focus on identifying recurring medicinal chemistry strategies and emerging structure–activity relationship (SAR) trends. Particular attention was given to the influence of structural features such as substitution patterns, degree of cationic character, molecular flexibility, and lipophilicity on biological activity and selectivity. The results were organized according to disease type and chemical class to provide a coherent and comparative overview of guanidine-based compounds across different neglected diseases.

SAR analysis was performed qualitatively and by scaffold class. A formal quantitative SAR comparison or meta-analysis was not performed because the included studies used heterogeneous chemical structures, heterogeneous biological models, parasite species or strains, developmental forms, assay endpoints, concentration units, exposure times, and cytotoxicity models. Therefore, SAR conclusions were interpreted as scaffold-specific tendencies rather than universal structure–activity rules.

## 4. Conclusions

Neglected diseases caused by protozoan parasites remain a major global health challenge, particularly in low- and middle-income regions where access to effective treatment and vector control strategies is limited. Within this context, the studies compiled in this review highlight the guanidine, guanidine-containing, guanidine-based compounds and related biguanides or amidinepharmacophores as a versatile and biologically privileged structural motif for the discovery of new antiprotozoal agents. Across different disease models, including schistosomiasis, Chagas disease, human African trypanosomiasis, schistosomiasis, tuberculosis and leishmaniasis, the presence of guanidinic, amidinic, or biguanidinic fragments repeatedly emerges as an important characteristic for biological activity, either through direct antiparasitic effects or through interference with key biochemical pathways involved in parasite survival and/or in the host–parasite interaction.

From a medicinal chemistry perspective, the collected evidence reveals several trends in the structure–activity relationship (SAR), which may contribute to the drug design process.

First, the cationic character of guanidinium groups favors electrostatic and hydrogen-bond interactions with biological targets such as enzymes, nucleic acids, and membrane components. This feature is particularly relevant, for example, in trypanosomatids, where guanidinium-containing molecules can interact with DNA minor grooves, metabolic enzymes, or redox pathways essential for parasite viability. Certain aromatic dicationic or polycationic compounds with appropriate geometry and charge distribution, such as diamidines (e.g., pentamidine-like compounds), may interact with AT-rich DNA minor grooves or kinetoplast DNA.

Second, poly-guanidinium architectures or dicationic systems may improve bioactivity through the enhancement of electrostatic interactions, membrane association, and target engagement in selected systems. However, these same properties can also increase cytotoxicity, reduce selectivity, impair passive permeability, compromise oral bioavailability, and limit clinical translation. Therefore, future optimization should aim to balance cationic character with drug-like physicochemical properties and experimentally validated mechanisms of action.

Third, peripheral substituents, such as halogens, aromatic heterocycles, or lipophilic fragments, play an important role in modulating lipophilicity, pKa, conformational effects, electronic distribution, membrane permeability, and target affinity. In several studies, fluorinated, chlorinated, or lipophilic substitutions improved potency, whereas excessive polarity often limited in vivo performance due to pharmacokinetic constraints.

At the same time, the review also highlights important drug-development challenges associated with guanidine derivatives. Their high basicity, strong ionization under physiological conditions, and sometimes limited membrane permeability may compromise oral bioavailability and tissue distribution. Consequently, future optimization strategies should focus not only on improving potency but also on balancing physicochemical properties such as lipophilicity, solubility, and metabolic stability. Approaches including prodrug design, scaffold hybridization, and rational modification of substituent patterns may help overcome these limitations.

Another important observation emerging from recent studies is the growing integration of multidisciplinary approaches in antiprotozoal drug discovery. Virtual screening, molecular docking, enzymatic assays, and phenotypic screening models have collectively accelerated the identification of promising guanidine-based candidates. In addition, strategies such as drug repositioning and vector-targeted interventions expand the therapeutic landscape beyond conventional antiparasitic chemotherapy.

Overall, the findings summarized in this review reinforce that guanidine, guanidine-containing, guanidine-based, and related biguanides or amidine motifs constitute a strategically valuable chemical class for the development of new therapies against neglected parasitic diseases, in selected scaffolds. However, their contribution is scaffold-dependent and should not be interpreted as a universal determinant of antiparasitic or antimicrobial activity. Continued exploration of their structural diversity by rational drug design, combined with advances in computational chemistry, molecular biology, molecular parasitology, and pharmacokinetic optimization, is likely to yield new candidates capable of addressing the limitations of current treatments. In this sense, guanidine-based scaffolds represent a promising yet insufficiently explored chemical space for the design of next-generation antiparasitic agents with improved efficacy, selectivity, and clinical potential.

In summary, this review highlights the chemical versatility of guanidine-, guanidinium-, guanidine-containing, and guanidine-related scaffolds (as amidines, biguanides, etc.) in the discovery of compounds active against neglected and emerging infectious diseases. Rather than acting as universal pharmacophores, these motifs contribute to biological activity in a scaffold- and context-dependent manner by modulating charge distribution, hydrogen-bonding capacity, lipophilicity, target recognition, and physicochemical behavior. However, clinical translation remains limited by recurrent challenges such as excessive polarity, poor oral absorption, limited membrane permeability, protonation-dependent trapping, potential efflux susceptibility, plasma protein binding, and toxicity associated with highly cationic systems. Future studies should therefore prioritize scaffold-specific optimization, balanced physicochemical properties, validated mechanisms of action, standardized biological assays, and integrated medicinal chemistry strategies combining synthetic design, computational modeling, pharmacokinetic evaluation, and in vivo efficacy assessment.

## Data Availability

No new data were created or analyzed in this study. Data sharing is not applicable.
